# Nickel and GTP Modulate *Helicobacter pylori* UreG Structural Flexibility

**DOI:** 10.3390/biom10071062

**Published:** 2020-07-16

**Authors:** Annalisa Pierro, Emilien Etienne, Guillaume Gerbaud, Bruno Guigliarelli, Stefano Ciurli, Valérie Belle, Barbara Zambelli, Elisabetta Mileo

**Affiliations:** 1 Aix Marseille Univ, CNRS, BIP, Bioénergétique et Ingénierie des Protéines, IMM, Marseille, France; apierro@imm.cnrs.fr (A.P.); eetienne@imm.cnrs.fr (E.E.); ggerbaud@imm.cnrs.fr (G.G.); guigliar@imm.cnrs.fr (B.G.); belle@imm.cnrs.fr (V.B.); 2 Laboratory of Bioinorganic Chemistry, Department of Pharmacy and Biotechnology, University of Bologna, 40127 Bologna, Italy; stefano.ciurli@unibo.it

**Keywords:** intrinsically disordered proteins, EPR spectroscopy, isothermal titration calorimetry, protein-ligand interaction, site-directed spin labeling, protein structural dynamics

## Abstract

UreG is a P-loop GTP hydrolase involved in the maturation of nickel-containing urease, an essential enzyme found in plants, fungi, bacteria, and archaea. This protein couples the hydrolysis of GTP to the delivery of Ni(II) into the active site of apo-urease, interacting with other urease chaperones in a multi-protein complex necessary for enzyme activation. Whereas the conformation of *Helicobacter pylori* (*Hp*) UreG was solved by crystallography when it is in complex with two other chaperones, in solution the protein was found in a disordered and flexible form, defining it as an intrinsically disordered enzyme and indicating that the well-folded structure found in the crystal state does not fully reflect the behavior of the protein in solution. Here, isothermal titration calorimetry and site-directed spin labeling coupled to electron paramagnetic spectroscopy were successfully combined to investigate *Hp*UreG structural dynamics in solution and the effect of Ni(II) and GTP on protein mobility. The results demonstrate that, although the protein maintains a flexible behavior in the metal and nucleotide bound forms, concomitant addition of Ni(II) and GTP exerts a structural change through the crosstalk of different protein regions.

## 1. Introduction

The discovery of antimicrobials to defeat bacterial pathogens is among the most important medical advances of the last century. However, antimicrobial resistance (AMR) has impaired the efficacy of antibiotics against infections in the last decades, and is considered by the World Health Organization (WHO) as one of the most important threats to public health for the next future [1,2]. In 2017, WHO listed the twelve most important resistant bacteria at a global level for which there is urgent need for new therapies [3]. Ten of them produce the virulence factor urease, a nickel-enzyme that hydrolyzes urea to produce ammonia and carbamate, thus leading to pH increase [4]. This event provides a suitable environment for host colonization, both by producing a micro-environment compatible with bacterial growth and by supplying nitrogen sources. For example, *Staphylococcus aureus* urease activity determines biofilm formation and is required for bacterial persistence [5,6], while for *Proteus mirabilis* [7], *Staphylococcus saprophyticus* [8] and *Ureaplasma ureolyticum* [9] urease activity plays a central role for infection and urea stones formation in the urinary tract. Several of these pathogens are involved in bacterial infections of the respiratory apparatus. It is remarkable that half of patients who died of the recent CoViD19 epidemics in Wuhan (China) became co-infected with bacteria in the lungs and also required antibiotics [10]. Therefore, urease is an attractive target for the development of innovative antibacterial molecules, acting both as antibiotics, as well as preventive anti-virulence drugs or adjuvants for bacterial eradication.

One of the best-known pathogens that exploits the enzymatic activity of urease is the Gram-negative bacterium *Helicobacter pylori*, a widespread microbe, infecting the stomach of up to 50% and 80% of adults in industrialized and developing countries, respectively [11]. The infection causes chronic inflammation of the gastric mucosa, which can slowly progress to gastric ulcer and, through the premalignant stages of atrophic gastritis, to gastric adenocarcinoma or gastric mucosa-associated lymphoid tissue (MALT) lymphoma. In 1994, the WHO classified *H. pylori* as a class I carcinogen. The neutralization of pH driven by urease is required by *H. pylori* for the colonization of the gastric niche [12], while the generated ammonia and induced platelet activation also plays a critical role in the inflammatory response of the host and in the progress of the disease [13].

Bacterial ureases are generally heteropolymeric proteins with a quaternary structure (αβγ)_3_ [4,14,15]. In the genus *Helicobacter*, the trimer is of the type (αβ)_3_, with the β subunits corresponding to the fused β and γ subunits normally found in other bacteria. The protein also presents a higher level of oligomerization with a [(αβ)_3_]_4_ quaternary structure [16]. Despite the different oligomeric organization, the structure of the known urease enzymes is fully conserved, and they present a substantially identical active site found in the α subunit. This site contains two Ni(II) ions bridged by the carboxylate group of a carbamylated lysine, essential to maintain the ions at the correct distance for catalysis, and by a hydroxide ion, the nucleophile in the hydrolysis reaction [4,14,15].

Although previous studies identified several molecules that bind urease and inhibit it competitively or uncompetitively, none of them is generally used in therapy, due to their severe side effects or limited ability to pass the bacterial membrane [14,15]. Recently, an alternative strategy to design urease inhibitors has been proposed by targeting, instead of the enzyme, the process that delivers nickel ions into the enzyme active site, precluding enzyme maturation to the active Ni(II)-loaded urease [17]. This activation process is governed by the interplay of at least four accessory proteins, named UreD, UreE, UreF, and UreG, coded by genes belonging to a single operon together with the structural genes [4]. UreE acts as the metallo-chaperone of the system that delivers Ni(II) into urease [18], through tunnels that pass across a complex formed by UreD, UreF, and UreG, the last acting as a molecular chaperone that prepares urease to incorporate the metal ion [19]. Precluding urease maturation by blocking delivery of Ni(II) into its active site could thus represent a novel approach to enzyme inhibition.

The central player of the urease chaperone activation network is UreG, a GTPase that couples the energy obtained from GTP hydrolysis to urease maturation [20]. *Hp*UreG interacts either with *Hp*UreE, forming a heterodimeric *Hp*UreG_2_E_2_ complex [18], or with *Hp*UreF and *Hp*UreD, forming a ternary *Hp*UreG_2_F_2_D_2_ complex [21]. The multiplicity of partners of UreG is reflected in its folding flexibility: while the structure of the protein has been reported for the GDP-bound *Hp*UreG in the *Hp*UreG_2_F_2_D_2_ complex [21], and for the GMPPNP (guanylyl imidodiphosphate)-bound *Klebsiella pneumonia* (Kp) UreG [22], in solution, both *Hp*UreG and *Kp*UreG feature high flexibility in solution, as shown by NMR spectroscopy, suggesting that the single conformation determined by X-ray crystallography does not reflect the flexible behavior of the protein in solution. This behavior is more generally observed in ^1^H,^15^N-HSQC NMR spectra of a plethora of UreG homologues from bacteria, archaea, and plants, which show broad signals with limited spread in the ^1^H dimension, indicating a backbone mobility in the intermediate exchange regime [23,24,25,26]. Native mass spectrometry and site-directed spin labeling coupled to electron paramagnetic resonance (SDSL-EPR) confirmed the presence of a heterogeneous conformational landscape for *Sporosarcina pasteurii* (*Sp*) UreG in the gas phase and in solution respectively, with at least two conformers with different degree of folding that coexist in equilibrium [27,28].

The selection of the binding partner is defined by the nucleotide bound state of the protein: GTP binding facilitates the formation of the *Hp*UreG_2_E_2_ [29], while the GDP-bound form preferentially interacts in the *Hp*UreG_2_F_2_D_2_ complex [21]. In addition, the concomitant presence of Ni(II) and GTP drives UreG dimerization in solution [21]. Ni(II) binds to a conserved Cys-Pro-His (CPH) motif, located on the protein interaction surface [30], while GTP binds on the opposite side of the protein [29].

A comparison of the crystal structure of the GMPPNP-bound *Kp*UreG and the GDP-bound UreG in the *Hp*UreG_2_F_2_D_2_ complex suggested that the presence of GTP drives an allosteric modulation of the Ni(II) binding site, which assumes a square planar geometry able to accommodate Ni(II) [22], suggesting that the two protein regions that bind Ni(II) and GTP/GDP communicate by allostery to drive the necessary conformational changes for UreG to function. However, such allosteric effect has not been proven in solution. The present study addresses this point, combining multiple biophysical approaches: SDSL-EPR, isothermal titration calorimetry (ITC), and static and dynamic light scattering (MALS-QELS).

In particular, we targeted three different regions of the protein with nitroxide-based spin labels and we performed both continuous wave and pulsed EPR spectroscopy in the presence of Ni(II) and GTP in order to determine their effect on the structural dynamics of *Hp*UreG in solution, as well as to investigate the structural crosstalk of different protein regions occurring by flexibility modulation. The results obtained were complemented by ITC and MALS-QELS. Altogether, the results show that the concomitant addition of both Ni(II) and GTP induces a modification of the structure and mobility in two regions of the protein.

## 2. Materials and Methods

### 2.1. Protein Expression and Purification

The purification of *Hp*UreG and its mutants was performed using a protocol previously reported [20]. We improved the yield of the protein expression growing the cells into auto-induction medium containing glycerol (5 g/L), glucose (25 g/L), and lactose (100 g/L), instead of LB combined with IPTG induction used in the previous work. The cells were grown 3 h at 37 °C and 18 h at 28 °C. At the last step of purification, the proteins were in 20 mM TrisHCl pH 8 buffer, containing NaCl 150 mM and TCEP 1 mM. Protein concentration was estimated using absorbance at 280 nm and an extinction coefficient ε_280_ = 10,032 M^−1^cm^−1^.

### 2.2. Isothermal Titration Calorimetry

Ni(II) binding titrations of wild-type and C66A mutant HpUreG were performed at 25 °C using a high-sensitivity VP-ITC microcalorimeter (MicroCal, Norcross, GA, USA). The protein and the metal ion salt (NiSO_4_) were diluted to 40–80 µM and 1.0 mM respectively into a solution of 20 mM TrisHCl pH 8, containing 150 mM NaCl and 1 mM TCEP, in the absence or in the presence of 150 µM of the non-hydrolyzable GTP analogue, GTPγS. A reference cell was filled with deionized water. Before each experiment, the baseline stability was verified. An interval of 5 min was applied between the injections to allow the system to reach thermal equilibrium. Control experiments were conducted by titration of the metal ion solution into the buffer alone under identical conditions, and the heat of dilution was negligible. The solution containing the protein was loaded into a sample cell (1.4093 mL) and was titrated with 55 × 5 µL injections with the Ni(II) solution. The raw data were processed and fitted using Affinimeter software, with a nonlinear least-squares minimization algorithm to theoretical titration curves with stoichiometric binding schemes. For Ni(II) titration over apo-HpUreG and C66A mutant, restriction of the binding parameters had to be made by fixing the stoichiometry of 2 and 1 respectively, as the low affinity binding did not provide an optimal sigmoidicity of the curve with a clear inflection point. Attempts to fit with stoichiometry of 1, 2, 3, and 4 were made, and the chosen stoichiometry was the one that provided the best fit of the experimental data.

### 2.3. HpUreG Mutants Design

The cysteine mutations were introduced into *Hp*UreG gene from Hp26695 strain urease operon (NCBI code NC000915) cloned into the pET15b expression vector (Novagen, Madison, WI, USA) in a previous work [20].

In order to relate and compare this work with previous studies, Cys66, wen mutated, was replaced by Alanine [18,21,29]. We decided to perform a Cysteine–Serine mutation for position 48 to preserve the surface charge of the protein, and a Cysteine–Alanine mutation in position 7 which is more buried in the crystal structure [22].

Mutants containing a single cysteine available for labeling were obtained by double mutation (“Site-directed mutagenesis” in Supplementary Materials Section S3). They were named as following: *Hp*UreG-C7A-C48S: C66^proxyl^; *Hp*UreG-C7A-C66A: C48^proxyl^; HpUreG-C66A-C48S: C7^proxyl^.

Variants containing two labeling sites and thus needing only one cysteine mutated were designed to perform distances measurements by DEER-EPR (“Site-directed mutagenesis” in Supplementary Materials Section S3) were named as following: *Hp*UreG-C7A: C48^proxyl^ /C66^proxyl^; *Hp*UreG-C66A: C7^proxyl^/C48^proxyl^; *Hp*UreG-C48S: C7^proxyl^/C66^proxyl^.

### 2.4. GTP Hydrolase Activity Assays

*Hp*UreG GTP hydrolyzing activity was measured by the SensoLyte^®^ MG Phosphate Assay Kit (AnaSpec, Fremont, CA, USA), based on the colorimetric reaction involving malachite green reagent, molybdate, and orthophosphate.

Each sample was prepared mixing the reagents in order to obtain 20 µM of protein, 400 µM of GTP, and 2 mM of MgSO_4_ in a final volume of 250 µL of buffer. The reaction mixture (RM) was incubated for 2 h at 37 °C. Every 30 min, 40 µL from the RM were incubated with 40 µL of Malachite Green Mix for 10 min in a final volume of 300 µL of buffer. After incubation, the absorbance at 600 nm was recorded. All the experiments were reproduced two times before estimating the Kcat values.

### 2.5. Protein Labeling with Nitroxide Spin Label

As *Hp*UreG variants are purified in presence of TCEP 1 mM, before labeling reaction, in order to avoid the reduction of the nitroxide spin label, the reductant removal is necessary. In general, a gel filtration using a PD-10 desalting column (GE Healthcare, Chicago, IL, USA) is sufficient. The labeling procedure is normally performed on 100 nmol of protein in a reductant-free buffer (Tris 20 mM, pH = 8, NaCl 150 mM) in the presence of a 10-fold excess of nitroxide spin label, the maleimido-Proxyl (Sigma-Aldrich, St. Luis, MO, USA). A 20-fold excess was used for double Cys variants. The mixture is then incubated at 4 °C, in the dark for 4 h under gentle stirring and continuous flow of argon. The excess of unbound label is removed by a second gel filtration with a PD-10 desalting column. The labeled protein is concentrated by using ultrafiltration (Vivaspin 5 kDa, Sartorius, Göttingen, Germany). The concentration of the labeled protein is evaluated by measuring the OD at 280 nm. The labeling yield of mono-labeled variants analyzed was between 80% and 100%, 150–170% for double-labeled ones.

### 2.6. EPR Spectroscopy

X-band room temperature (298 K) continuous wave EPR measurements were recorded on an Elexsys500 Bruker spectrometer equipped with a Super High Q sensitivity resonator operating at X band (9.9 GHz). The microwaves power was 10 mW, the magnetic field modulation amplitude was 0.1 mT, the field sweep was 15 mT, the receiver gain was 60 dB. All the samples were analyzed in quartz capillaries whose sensible volume was 40 µL.

The spin concentration was obtained by double integration of the EPR signal obtained under non-saturating conditions and the labeling yield was evaluated comparing the spin concentration with that one of a standard solution. For all variants, high labeling yields were obtained ranging from 80% to 100% for mono labeled samples and 150–170% for double-labeled samples.

X-band cw EPR spectra at room temperature were recorded at 50 µM of protein concentration in Tris 20 mM, pH = 8, NaCl = 150 mM. When present, Ni(II) was 2.5 mM (NiSO_4_), GTP/GDP 3 mM (Sigma-Aldrich).

The EPR spectra were simulated using SimLabel program [31], a Matlab graphical user interface using the Easyspin toolbox [32].

### 2.7. DEER Measurements

Inter-label distance distributions were obtained using the four-pulse DEER sequence [33]. Experiments were performed on a Bruker ELEXSYS E580 spectrometer at Q-band using the standard EN 5107D2 resonator. The system was equipped with an Oxford helium temperature regulation unit and the data were acquired at 60 K. This temperature was optimized according to the relaxation times measured at variable temperatures in the range of 20–100 K with 10 K steps. All the measurements were performed on 20 µL of sample loaded into quartz capillaries. DEER samples were flash frozen in liquid nitrogen. Distance distribution were extracted from DEER data through a Tikhonov regularization after baseline correction, using DeerAnalysis2019 software (http://www.epr.ethz.ch/software/index Jeschke G. 2011. DeerAnalysis. ETH, Zürich, Switzerland). Distance distributions measured were compared with the distance distributions predicted, analyzing the crystal of HpUreG (PDB 4HI0) [22] using the MMM software [34].

## 3. Results and Discussion

### 3.1. Cys Variants Were Generated to Selectively Label Distinct Regions of HpUreG

SDSL-EPR spectroscopy is a non-destructive technique that provides details on protein structure and flexibility over a wide-range of temperatures and timescales [35,36,37,38]. Proteins can be studied in their native environment, that is in membranes, in cellular extract and also inside cells [39]. SDSL-EPR involves the grafting of a paramagnetic label, generally a thiol-specific nitroxide, on the protein of interest and the determination of the dynamic properties of the attached nitroxide by continuous wave (CW)-EPR spectroscopy [40,41]. Changes in the nitroxide spectrum are thoroughly related to the mobility of the nitroxide side-chain and to the local backbone motion, which can thus be used to follow protein structural changes and to reveal interaction sites in complexes in solution and at room temperature [40,42,43,44,45]. Distance distributions between two spin labels can be measured by pulsed double electron–electron resonance (DEER) techniques relying on their dipole–dipole coupling [46,47]. Inter-label distance distributions can be investigated between 15 and 80 Å, but in specific experimental conditions, 160 Å can be reached [48]. DEER experiments are usually carried out at cryogenic temperature (60 K), the low temperature being required to slow down the otherwise fast relaxation of nitroxide labels at higher temperature. As for all the other techniques requiring a freezing step, it is assumed that the conformational ensemble of the sample is captured.

Since most of the available nitroxide-based spin labels can specifically react with the thiol group of Cysteine, site-directed mutagenesis is often used to introduce Cys residues at specific locations in the primary structure of the protein of interest.

**Figure 1:**
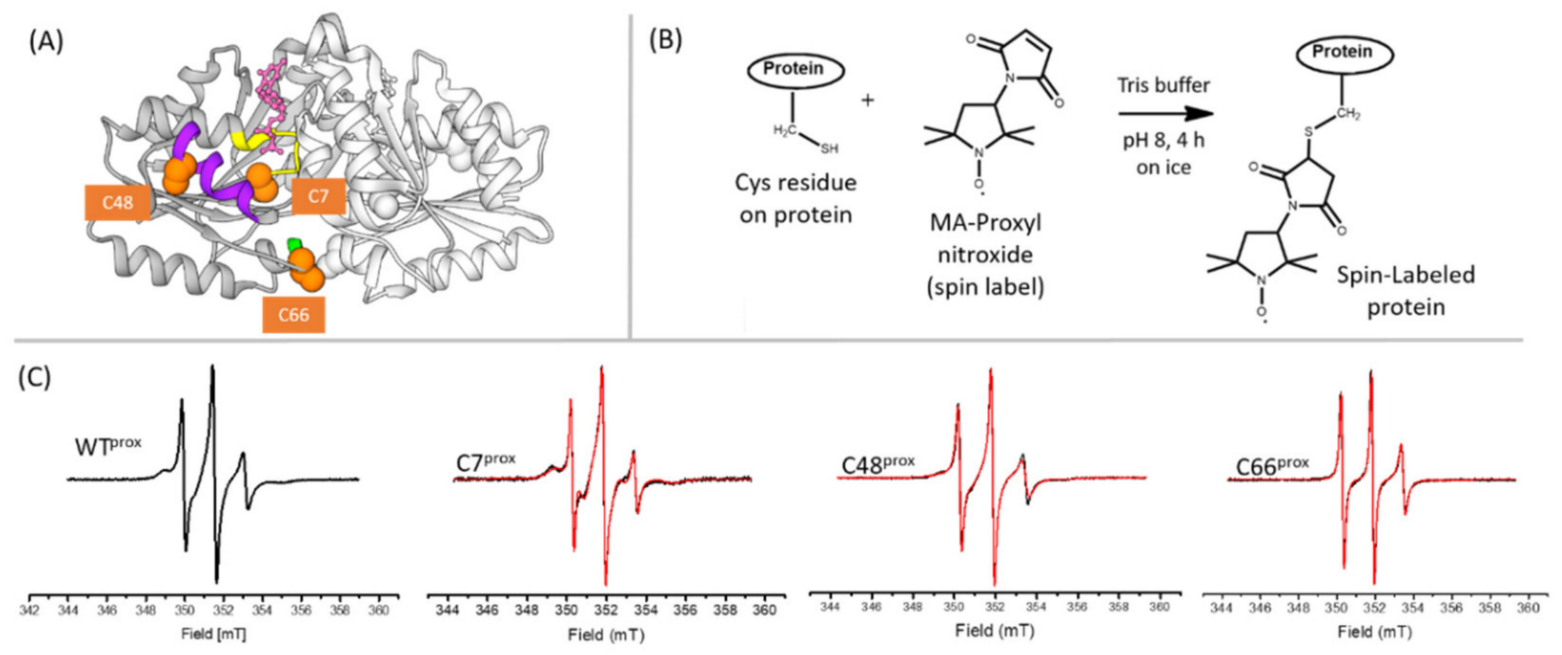
(**A**) *Hp*UreG structure in the presence of GDP (pink) from the *Hp*UreG_2_F_2_D_2_ crystal (PDB 4HI0). The position of the three natural cysteine residues is highlighted in orange: Cys7 in the P-loop (yellow), Cys48 in the Helix 2 (purple), and Cys66 in the CPH Nickel binding site (green). (**B**) Labeling reaction scheme with MA-PROXYL nitroxide label. (**C**) Room temperature X-band EPR spectra of 50 µM of wild-type *Hp*UreG and its variants labeled with MA-PROXYL nitroxide in Tris buffer (black trace) and superimposed simulated spectra (red traces).

*Hp*UreG has three naturally occurring Cys, located in different regions of the protein (Figure 1A): (i) the conserved P-loop-motif, involved in GTP binding [20], accommodates Cys7; (ii) Helix 2, involved in GTP-dependent conformational changes, contains Cys48 [21]; (iii) the fully conserved CPH motif, involved in Ni(II) binding, includes Cys66 [20]. These positions allow, in principle, to monitor the mobility of three functionally important regions of the protein in solution. Consequently, six mutants containing one or two Cys residues were designed and labeled with the MA-Proxyl nitroxide to dissect the protein conformational landscape (Figure 1B): three double variants feature a unique position available for labeling (C7^prox^, corresponding to the Cys48Ser/Cys66Ala labeled mutant; C48^prox^ corresponding to the Cys7Ala/Cys66Ala labeled mutant; C66^prox^, corresponding to the Cys7Ala/Cys48Ser labeled mutant), while three single variants possess two positions available for labeling for distance measurements (C7^prox^/C48^prox^, corresponding to the Cys66Ala labeled mutant; C7^prox^/C66^prox^, corresponding to the Cys48Ser labeled mutant; C48^prox^/C66^prox^, corresponding to the Cys7Ala labeled mutant). Note that “WT^prox^” corresponds to the wild-type protein (WT) labeled in the three naturally occurring Cys residues. The labeling reactions were checked by mass spectroscopy (see Supplementary Materials Figure S1). Any possible perturbation of the global structure and of the folding of *Hp*UreG mutations was excluded by controlling the global folding by circular dichroism (CD, see Figure S2). Similarly, the catalytic activity of *Hp*UreG was monitored for the wild-type protein labeled in the three Cys positions (WT^prox^) (see Figure S1A), indicating no significant differences between the unlabeled and labeled proteins (*k_cat_* = 0.027 min^−1^ and *k_cat_* = 0.023 min^−1^, respectively).

### 3.2. The Thermodynamics of Ni(II) and GTP-Driven Dimerization of HpUreG Was Characterized

Previous studies of Ni(II) binding to *Hp*UreG entailed the use of ITC, which showed that the isolated protein interacts with two Ni(II) ions per monomer with an exothermic reaction and a dissociation constant *K_d_* = 10 µM [20]. Ni(II) binding was also monitored using the gradual increase of absorption peak at 337 nm, assigned to ligand-to-metal-charge transfer [29]. Differently from the ITC experiments, the latter approach did not detect any Ni(II) binding activity for the isolated protein, whereas metal binding occurred when GTP was added to the protein solution, under which condition *Hp*UreG was found to bind 0.5 equivalents of Ni(II) per protein monomer, with *K_d_* = 0.33 µM, and to undergo dimerization upon metal binding [29].

**Figure 2:**
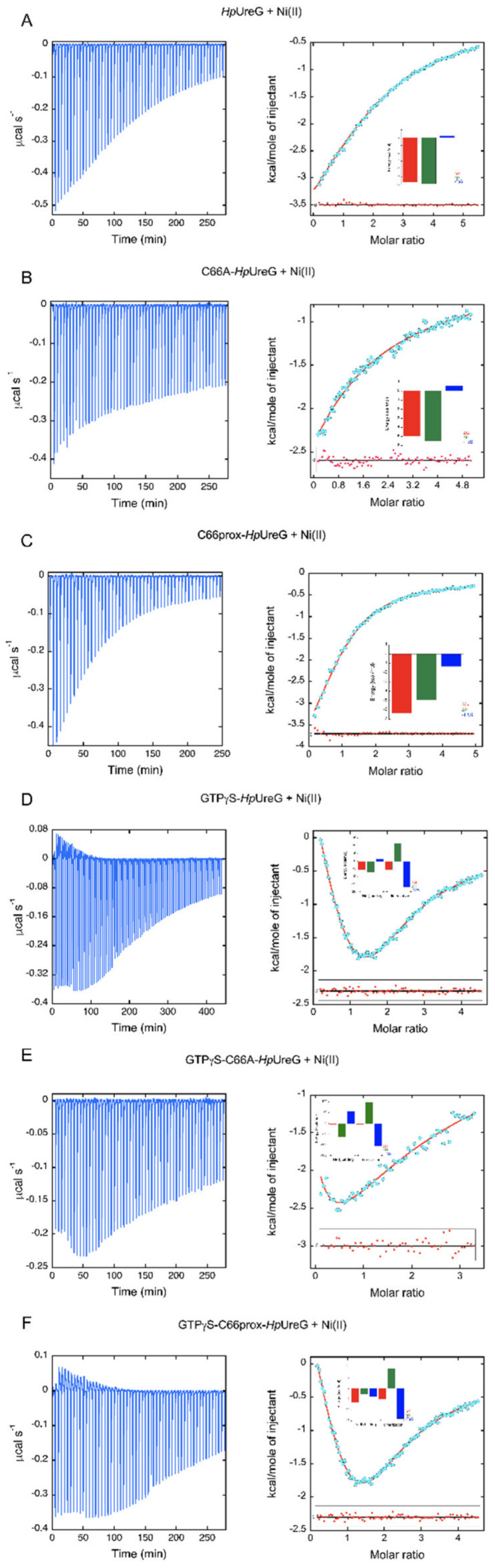
Isothermal Titration Calorimetry (ITC) of Ni(II) over *Hp*UreG and its variants in the presence and in the absence of GTPγS in the sample cell. The panels on the left report the heat flow response for the injections of the metal ion over the protein solutions. The panels on the right show the respective integrated heat data (blue circles) and their best fit obtained using a single set of binding sites (**A**–**C**) or a model involving protein dimerization upon metal binding (**D**–**F**); the insets show the binding signatures (ΔG, ΔH, −ΔT, ΔS) associated to each curve; the bottom plots show the residuals of the fitting procedure.

Calorimetric titration of Ni(II) over a freshly purified *Hp*UreG sample, performed here for comparison with all other ITC data on *Hp*UreG mutants and labeled forms described in the present study, confirmed the results previously obtained by ITC, showing negative peaks following each metal additions, indicative of an exothermic binding event (Figure 2A, left panel). The integrated heat data generated a binding isotherm with a single inflection point, and a mild slope (Figure 2A, right panel and Table 1). The fit of the obtained data, performed using the AFFINImeter software [49] and a model involving a single set of sites, showed that two Ni(II) ions bind per *Hp*UreG monomer with similar affinity (*K_d_* = 72 µM), a favorable enthalpic contribution and a minor entropic impact (Table 1). Previously reported studies on *Hp*UreG mutants indicated that at least one Ni(II) binding site is located on the CPH motif [20,29]. These observations outline two possible scenarios: (i) both Ni(II) ions bind to identical sites located in the region of CPH, or (ii) one Ni(II) ion binds to the CPH motif while the second binds to the different site; in the latter case, a possibility is represented by the Mg(II) binding sites close to the GTP binding pocket, as previously suggested [29]. The difference in Ni(II) affinity thus measured for *Hp*UreG (72 µM) with the previously reported value obtained by ITC (10 µM) [20] is likely due to the experimental conditions: for the ITC titrations, the relatively weak metal–protein affinity caused the value of the c-parameter, namely the product of the concentration of the protein in the cell by the binding constant, to be close to its lowest acceptable limit [20], rendering the calculated affinity constants less accurate.

Mutation of the Ni(II) binding residue Cys66 to Ala was previously reported to fully abolish Ni(II) binding capability of the protein [29]. Here, the ITC titration instead revealed that the Cys66Ala-*Hp*UreG mutant is still able to bind one Ni(II) ion per protein monomer, with an exothermic reaction (Figure 2B, left panel) and one order of magnitude lower affinity (*Kd* = 236 µM), with substantially invariant enthalpic and entropic contributions (Figure 2B, right panel and Table 1). This observation suggests that the two Ni(II) binding sites per monomer, observed in wild-type *Hp*UreG, are distinct, with one site, involving Cys66 in the CPH motif, that is abrogated by the Cys66-to-Ala mutation, while the second is maintained. The decreased affinity for the latter site (*K_d_* = 236 µM vs. 72 µM for the mutated and WT protein, respectively) suggests the presence of cooperativity between the two metal binding sites. The triply labeled WT^prox^, while showing the same exothermic effect (Figure 2C, left panel) and the same stoichiometry as the Cys66Ala mutant, features an higher affinity for Ni(II) (*K_d_* = 23 µM), a similar enthalpic value and positive entropic contribution (right panel of Figure 2C and Table 1), as compared to the Cys66Ala mutant (*K_d_* = 236 µM), indicating that the Cys labeling with the nitroxide moiety still abolishes one of the two Ni(II) binding sites observed for the unlabeled WT protein, but, differently from the Cys66Ala mutation, maintains a similar affinity for the second Ni(II) binding event, supporting the idea of cooperativity between the two Ni-binding sites in the WT protein.

Ni(II)- and GTPγS -driven dimerization [29] was verified under the ITC experimental conditions using size-exclusion chromatography coupled to multi-angle light scattering (SEC-MALS, Figure S3). The obtained results confirmed that *Hp*UreG undergoes dimerization when both Ni(II) and GTPγS, a non-hydrolyzable GTP analogue, are added to the protein solution, while it remains monomeric in the presence of either Ni(II) or GTPγS alone.

**Scheme 1:**
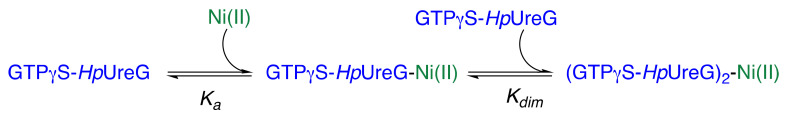
Equation defining the model used to fit the ITC data for Ni(II) titration over GTPγS-HpUreG, involving metal binding and protein dimerization.

The thermodynamics of Ni(II) and GTPγS-driven protein dimerization was therefore addressed using ITC. Ni(II) ions were titrated over *Hp*UreG in the presence of GTPγS in the sample cell. Negative peaks followed each injection of Ni(II) into the protein solution, indicating the occurrence of an exothermic reaction (Figure 2D, left panel). The observation of a slower endothermic effect following each injection, which terminates when one equivalent of metal is added to the protein solution, suggested the existence of another process, in addition to metal binding, similarly to what had been previously observed for the Ni(II)-sensor *Hp*NikR [50]. This type of ITC trace can be interpreted either as a conformational modification or as a change in the oligomerization state of the protein. As dimerization was demonstrated by light scattering experiments (Figure S3) [29], and the shape of the binding isotherm clearly indicated two inflection points suggestive of two reactions occurring upon metal titration, the data were analyzed using a model involving two successive equilibria, with protein dimerization following the binding of one Ni(II) per protein dimer (Scheme 1).

Fitting of the binding isotherm (Figure 2D, right panel and Table 1) indicated that one Ni(II) ion binds per protein monomer in the presence of GTPγS, with a dissociation constant two times smaller than that reported for the apo-protein (*K_d_* = 30 µM), while protein dimerization occurs with *K_dim_* = 71 µM. In this case, Ni(II) binding occurs with thermodynamic parameters similar to the ones observed for the apo-protein, with favorable enthalpy and a negative entropic contribution (Table 1). On the other hand, dimerization is an entropy-driven endothermic process as expected (Table 1). The entire two-step process is characterized by a global dissociation constant of *K_d_* = 2 nM. This value should be compared with that obtained by absorbance spectroscopy for Ni(II) binding to *Hp*UreG in the presence of GTPγS (*K_d_* = 0.33 µM) [29]. The difference between these two values could be attributed to the different pH at which the measurements were carried out in the present (8.0) and in the previous (7.2) work. The apparent decrease in affinity at lower pH is consistent with a proton dissociation event occurring upon metal binding, which possibly involves a cysteine residue (Cys66 in this case), as previously observed in the case of the nickel-dependent transcription factor *Hp*NikR [50].

Ni(II) titration over the Cys66Ala mutant in the presence of GTPγS produced negative peaks indicative of an exothermic binding of Ni(II), but no endothermic effect was visible (Figure 2E, left panel), suggesting that a dimerization is either not occurring in this case or is occurring with much lower affinity, resulting in the absence of detectable endothermic heat. The binding isotherm (Figure 2E, right panel) showed two inflection points, and the same model reported in Scheme 1 was used to treat the data. According to the fit, the first event of Ni(II) binding occurs with one order of magnitude lower affinity (*K_d_* = 160 µM) as observed for the Cys66Ala mutant in the absence of GTPγS (Figure 2A), while the second equilibrium shows a similar constant (*K_d_* = 22 µM). In this case, the thermodynamic parameters associated to the metal binding step and to the second process (Table 1) are unusually high compared to all other similar data in this study, suggesting that additional phenomena other than metal binding and dimerization are occurring in the case of this mutant. It is worth noticing that, during sample manipulation, the Cys66Ala mutant was prone to precipitation, especially in the presence of Ni(II), suggesting that at least part of the protein sample undergoes aggregation upon Ni(II) titration, which might be the second process evidenced in the binding isotherm.

Ni(II) titration over the triply labeled WT^prox^ protein in the presence of GTPγS (Figure 2F, left panel) produced a bipartite binding isotherm (Figure 2F, right panel), whose analysis, performed according to Scheme 1, indicated that Ni(II) ion binding to the protein dimer occurs with an affinity similar to the wild type protein (*K_d_* = 23.8 µM), and favorable enthalpic and entropic contributions (Table 1). On the other hand, the second process is less favorable for WT^prox^, occurring with a lower equilibrium constant (*K_d_* = 260 µM) as compared to the dimerization of the WT protein. If the second process also involves dimerization for WT^prox^ (as suggested by the values of *ΔH* and *ΔS*, see Table 1), this decreased value could be due to a steric effect of the nitroxide label.

### 3.3. HpUreG Shows Distinct Flexibility in Different Protein Regions

The EPR spectrum of WT *Hp*UreG labeled with MA-Proxyl nitroxide (WT^Prox^, Figure 1C) arises from the contribution of spin labels simultaneously grafted onto the three Cys residues. To separately dissect the conformational flexibility of different regions of *Hp*UreG, the EPR spectrum of the nitroxide-labeled *Hp*UreG variants that contain a single labeled cysteine (C7^prox^, C48^prox^, and C66^prox^) were performed in solution and at room temperature (Figure 1C). Qualitatively, when the nitroxide mobility decreases, a broadening of the EPR spectral line shape is expected. For the single labelled *Hp*UreG variants, the spectra show different mobility: the line shape becomes sharper going from C7^prox^ to C48^prox^ and then to C66^prox^, reflecting an increased mobility of the nitroxide moiety and, consequently, of the protein structural motif to which the label is attached (Figure 1C). A quantitative view of the nitroxide dynamics in terms of the rotational correlation time (τ_c_) and of the magnetic parameters (*g*-factor and hyperfine A-tensors) was obtained by simulating the EPR spectra with SimLabel [31] (a MatLab graphical user interface using the Easyspin toolbox [32]) (see Table 2 and Supplementary Materials Section S2). As very often found in SDSL-EPR studies [27], the spectra of *Hp*UreG could be simulated by two components, which represent populations of spin labels characterized by different dynamics. These populations can be related either to rotameric states of the spin label or to structural sub-states of the protein in conformational equilibrium. In the case of *Hp*UreG, the large difference in the dynamics of the two components (see τ_c_ in Table 2), generally not observed for rotamers [51], suggests that they reflect distinct protein conformational states [51,52]. This conclusion is consistent with similar phenomena reported for *Sp*UreG [27], and is further supported by the observation that the *Hp*UreG EPR spectrum of C66^prox^ is modified by the addition of glycerol, a protective osmolyte: in this case, the spectrum can be simulated by increasing the contribution of the slower component, which changes from 44 to 70% (Table 2 and Figure S4). Protective osmolytes are indeed known to modify the conformational equilibria among different conformational states of the protein [53], stabilizing the protein structure toward a more folded conformation [52].

**Figure 3:**
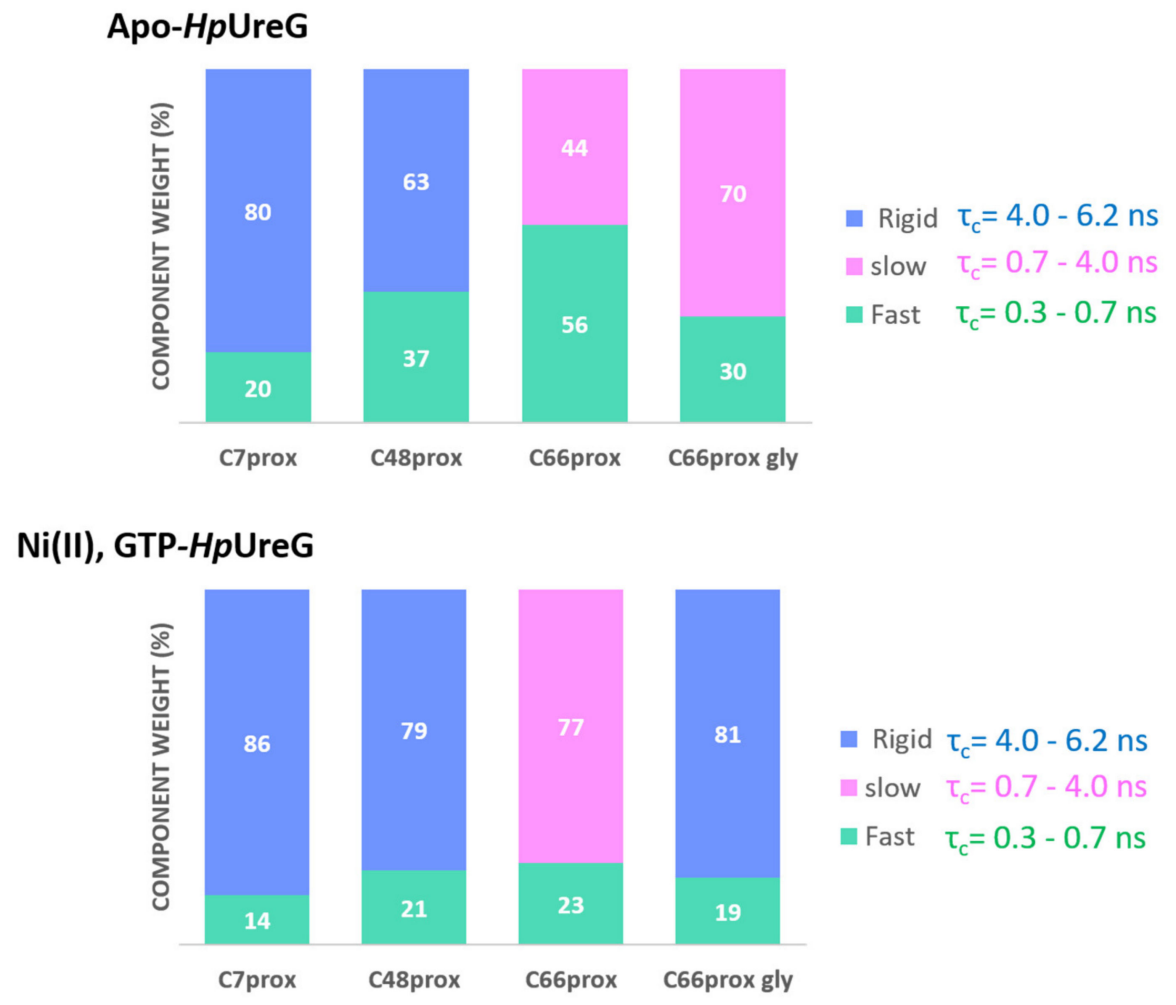
Conformational distribution of *Hp*UreG variants plotted in terms of the relative amount of the conformations obtained from simulation of EPR spectra. The different conformational ensembles are indicated using different colors and the relative correlation times are reported.

Note that, in the following paragraphs, we named “fast” all spectral components characterized by τ_c_ values included between 0.3–0.7 ns, “slow” those characterized by τ_c_ values included between 0.7–4.0 ns, and “rigid” components characterized by τ_c_ values included between 4.0–6.2 ns. The sharpest EPR line shape is observed for the nitroxide grafted to Cys66. This spectrum is constituted by two components having almost the same proportion, one with τ_c_ = 0.6 ns (“fast”) and the other with τ_c_ = 2.4 ns (“slow”) (Table 2 and Figure 3). The fact that the “fast” component shows a mobility close to that normally observed for a spin label attached to loops or intrinsically disordered protein fragments [27,44,54] demonstrates that this region is highly flexible. This dynamic behaviour is similar to that observed for the *Sp*UreG orthologue containing the nitroxide label grafted onto the corresponding cysteine residue, which features two conformers with similar correlation times (τ_c_ “fast” = 0.3 ns and τ_c_ “slow” = 3.6 ns) (see Figure S5) [27].

Similarly, two components with different degrees of flexibility and comparable relative abundance are observed for C48^prox^: one features a “fast” behavior (τ_c_ = 0.6 ns), while the other (“rigid”) is consistent with a less flexible dynamic (τ_c_ = 4.9 ns) (Table 2 and Figure 3). On the other hand, a “rigid” component (τ_c_ = 6.1 ns) is dominant (80%) in the case of C7^prox^, for which a less abundant component (20%) shows a “fast” behavior (τ_c_ = 0.3 ns) (Table 2 and Figure 3). The latter case can be explained by considering that the nitroxide moiety resides in a well-structured region or in a buried site. The high yield of labeling reached for this site (80–100%, Figure S1) suggests that Cys7 is accessible, so the observed rigid behavior is indicative of the presence of a highly rigid protein segment.

All these data experimentally confirm previously reported molecular dynamic simulations on *Hp*UreG, which suggested substantial rigidity in the protein regions involved in catalysis, justifying the residual catalytic activity of the isolated proteins, while evidencing the large dynamic flexibility for the protein portions involved in protein−protein interactions, which contain the residues in the conserved CPH motif [55].

**Figure 4:**
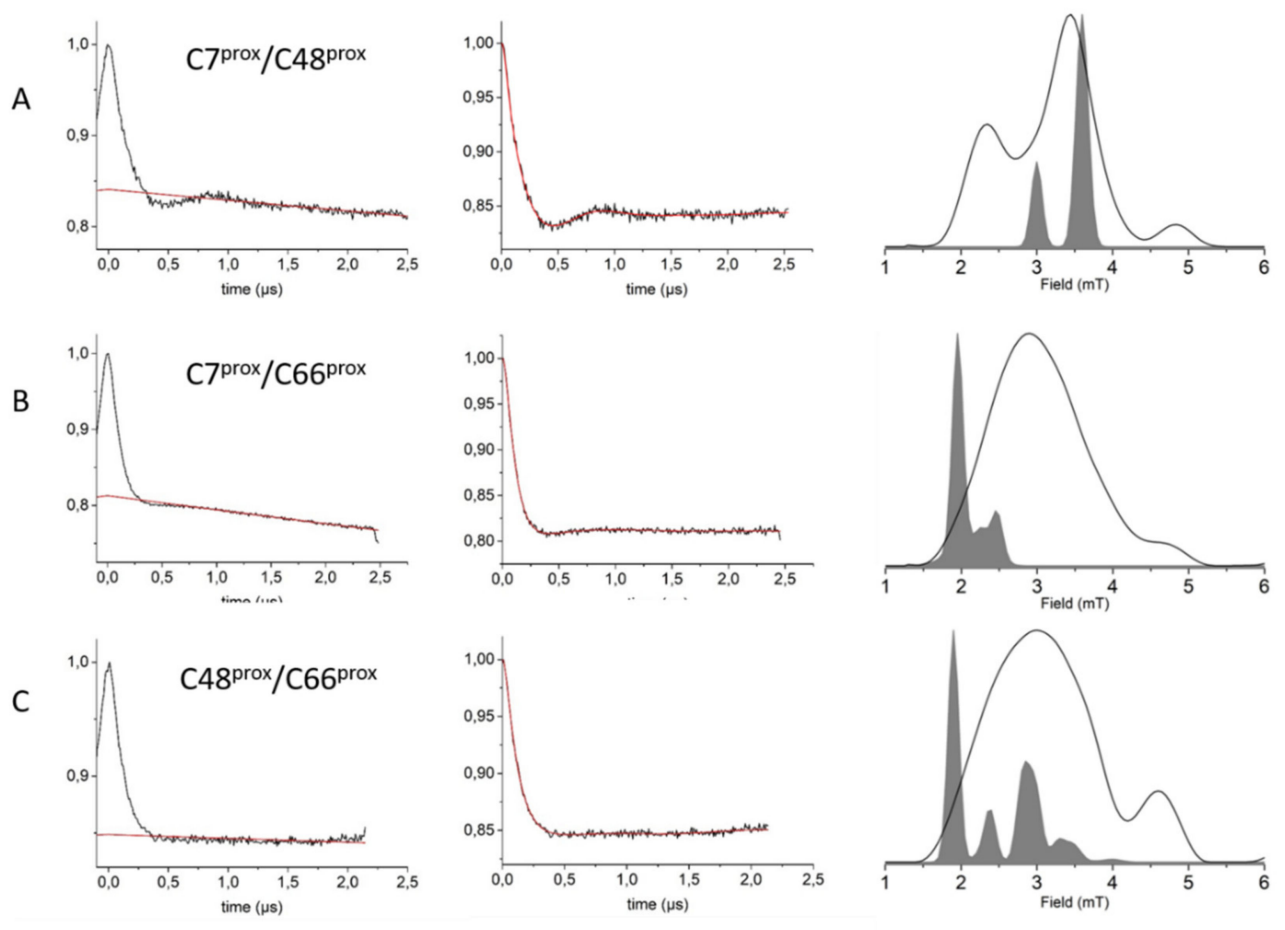
Inter-label distance distributions. (**Left panel**): experimental Q-band DEER traces recorded at 60 K for (**A**) C7^prox^/C48^prox^, (**B**) C7^prox^/C66^prox^, and (**C**) C48^prox^/C66^prox^. Red lines indicate the baseline used for background correction. (**Central panel**): corrected Double Electron-Electron Resonance (DEER) traces (black) with superimposed fits derived from Tikhonov regularization (red). (**Right panel**): Tikhonov derived distance distributions obtained using DeerAnalysis (black) [56] superimposed with distance distributions calculated by MMM software (gray curves) [34].

To further investigate the structural dynamics of *Hp*UreG in the apo-state, double electron-electron resonance (DEER) experiments were applied. DEER experiments allow to measure the dipolar coupling between spin pairs, yielding distance distribution between the coupled spins. Three double-Cys variants were constructed and labeled (C7^prox^/C48^prox^, C7^prox^/C66^prox^, C48^prox^/C66^prox^) and their CW EPR spectra are reported in Supplementary Materials Figure S7. For all the DEER data shown in this section, the error on distance distribution results was calculated with the validation tool of DeerAnalysis (see Figure S8) [56]. DEER data of the C7^prox^/C48^prox^ variant (Figure 4A) showed well-resolved distance distribution with 2 peaks centered at 2.4 and 3.5 nm, while the one obtained for C7^proxyl^/C66^proxyl^ (Figure 4B) and for C48^proxyl^/C66^proxyl^ (Figure 4C) displayed broad distance distribution. These results confirm the presence of considerable conformational heterogeneity in the protein sample, supporting the highly flexible behavior of *Hp*UreG, as already observed by previous NMR studies [20] and by the CW EPR data described above.

In all cases, the average distance distributions measured by DEER span a broader range than those predicted by an MMM [34] analysis based on the *Hp*UreG crystal structure (PDB: 4HI10) [21] and a library of rotamers for the MA-Proxyl spin label (Figure 4). This observation confirms that the experimental conditions used in the crystallization experiments (solutes and salts acting as precipitants) likely favored a more compact and rigid fold, as already shown for other biological systems [57], and that the protein structure observed in the crystal is different from the conformation that the protein assumes in solution.

### 3.4. Ni(II) Ions and GTP Binding Produce Changes in the Structural Dynamics of Different Protein Regions

**Figure 5:**
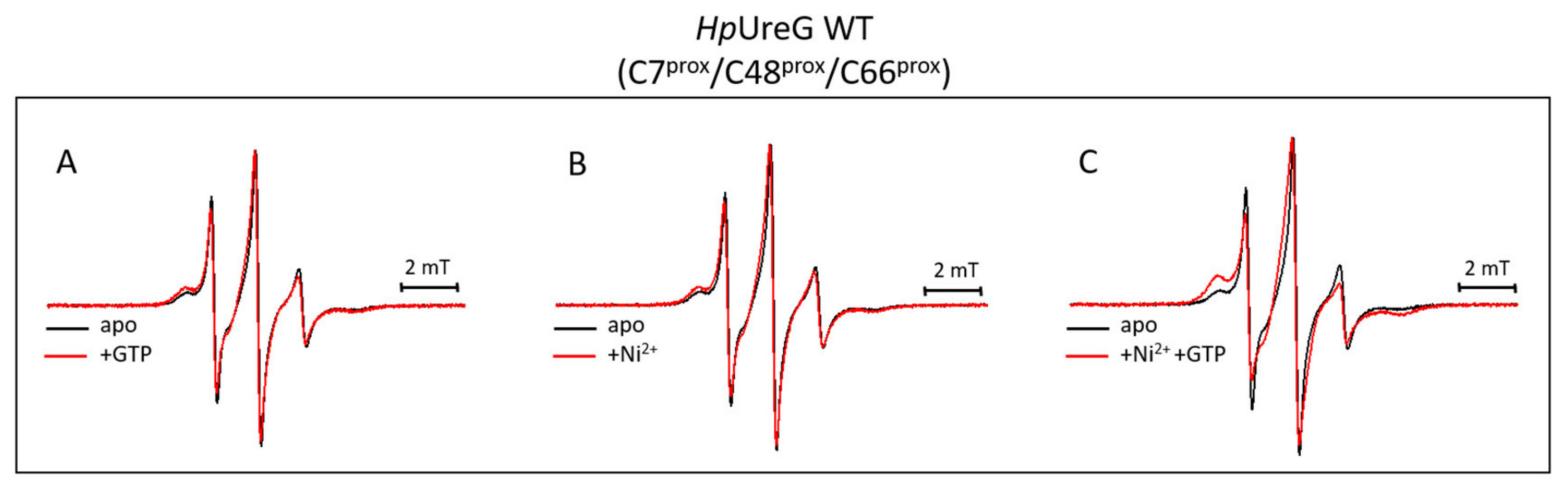
X-band CW EPR spectra of 50 µM *Hp*UreG labeled at the three naturally occurring Cys residues, recorded in solution and at room temperature. The apo-form spectra (black line) are compared to spectra recorded in presence of either 3 mM GTP and/or 2.5 mM Ni(II) (red line).

Figure 5 shows the EPR spectra of the WT^prox^ in the presence of either GTP or Ni(II), and in the presence of both cofactors. No significant spectral changes were detected after the addition of either GTP or Ni(II), while addition of both cofactors resulted in a clear change of the EPR line shape, corresponding to an increase of the broader components of the spectrum. This indicates that both ligands are necessary to induce structural changes in *Hp*UreG.

To investigate the source of this spectral modifications, and in particular to sort out which of the three nitroxide labels bound to the protein contributes to the Ni(II) and GTP-driven line broadening, the experiment was repeated using the single-Cys variants of *Hp*UreG.

**Figure 6:**
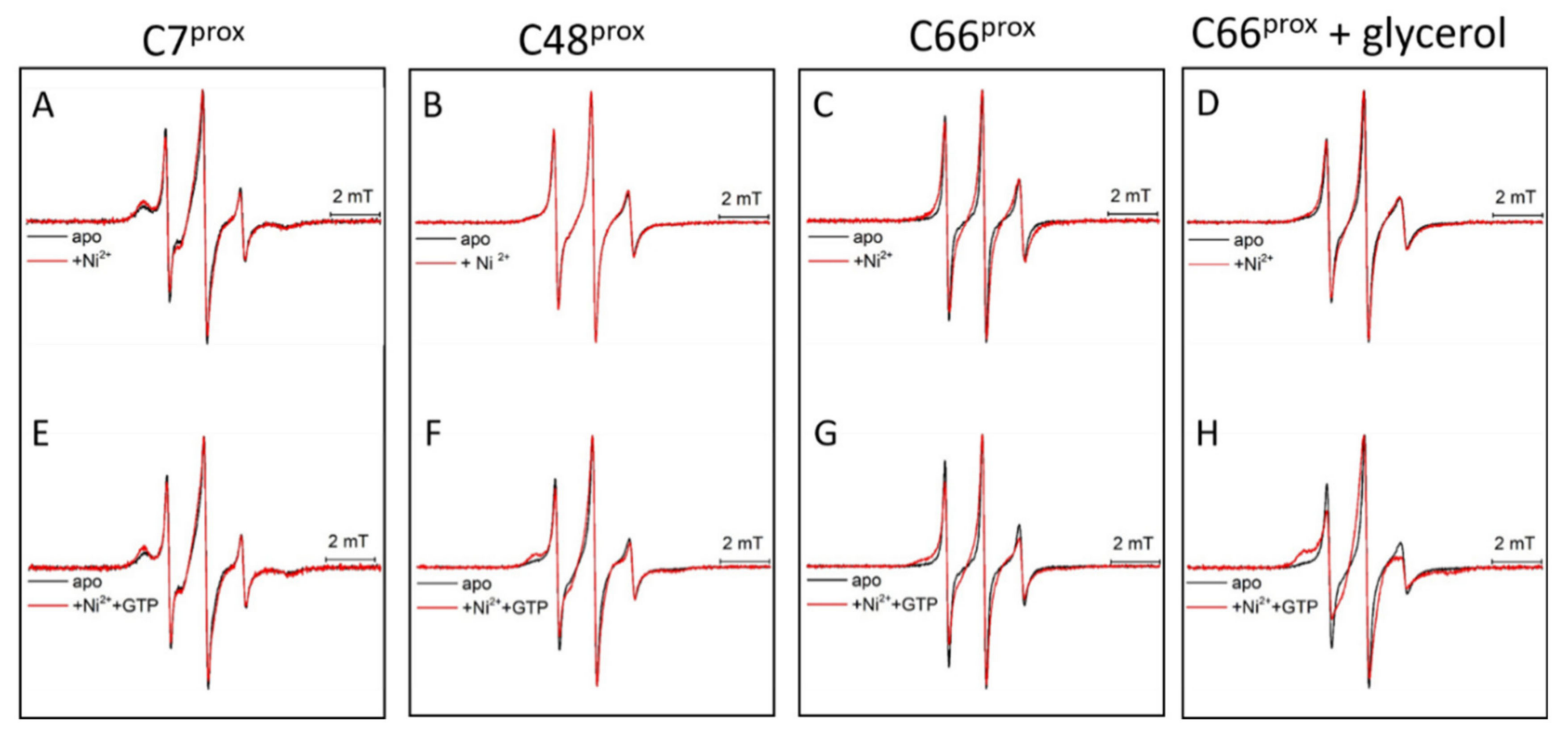
X-band CW EPR spectra of *Hp*UreG variants at room temperature, in the absence and in the presence of glycerol. In the upper panels (**A**–**D**), the apo-form spectra are in black, those obtained in presence of 2.5 mM of NiSO_4_ are in red. In the lower panels (**E**–**H**), the apo-form spectra (**black line**) are compared to spectra recorded in presence of either 3 mM GTP and/or 2.5 mM Ni(II) (**red line**).

Addition of Ni(II) did not produce significant changes of the EPR line shapes for C7^prox^ (Figure 6A) and C48^prox^ (Figure 6B), while for the C66^prox^ spectrum induced a line broadening (Figure 6C), suggesting a reduction of the spin label mobility (Table 2 and Figure 3). Indeed, we observe a change in the weight of the two components (Table 2 and Figure 3), with the less flexible species becoming most abundant (from 44 to 66%).

Differently, no significant spectral changes were detected on all protein variants after the addition of GTP or GDP alone (Figures S9 and S10). We also tested the effect of Mg(II) to protein samples containing GTP or GDP. The addition of Mg(II) in equimolar concentration with respect to GTP, did not affect the spectral shape of the protein (Supplementary Materials Figure S11).

Concomitant addition of both Ni(II) and GTP did not significantly affect the spectrum of C7^prox^ (Figure 6E), while it changed the line shape of C48^prox^ (Figure 6F), with a conformational shift toward the “rigid” component, which increased from 63% to 79% (Table 2 and Figure 3). In the case of C66^prox^, additional spectral changes were observed when GTP and Ni(II) bind to the protein; the τ_c_ of both components increases, suggesting an induced structuration of this protein region (Table 2 and Supplementary Materials Section S2) or the dimerization of the protein upon both GTP and metal binding. These changes became more pronounced if the experiments were performed in the presence of glycerol, with a drastic change in the dynamics of the less flexible specie (Figure 6H, red trace), whose τ_c_ varies from 2.4 ns to 4.4 ns (Table 2). These results were confirmed for the *Hp*UreG C48^prox^/C66^prox^ variant, which showed a similar behavior as found for C66^prox^ in glycerol (Figure S12).

The observed spectral changes indicate that Ni(II) and GTP induce structural and dynamics modifications in the Ni(II)-binding region of the protein where C66 is located, and in the region around Helix 2, containing C48. This suggests the occurrence of an allosteric communication between the protein regions proximal to the Ni(II) and the GTP binding sites, whereas the region around the Cys7 residue is not affected by the presence of either Ni or GTP, or both.

To further investigate the structural effect of ligand binding on *Hp*UreG fold, DEER experiments would have been of great interest. However, the decrease in the T_m_ (phase memory time) value (Figure S13A) associated with the field sweep (FS) intensity loss (Figure S13B) in the presence of Ni(II) prevented from obtaining properly exploitable DEER traces. Works are currently in progress to improve the quality of DEER experiments.

## 4. Conclusions

In this work, the structural dynamics of *Hp*UreG was explored in the absence and in the presence of its physiological cofactors, Ni(II) and GTP. In solution, *Hp*UreG fluctuates between different sub-states, this plasticity likely being a key factor to allow the protein to perform protein–protein and protein–metal ion interactions needed for Ni(II) ions delivery into the urease active site. ITC determined the conditions and the thermodynamic parameters of Ni(II) and GTP-driven protein dimerization, supported by light scattering data. SDSL-EPR demonstrated that the degree of structural flexibility changes along the protein backbone, with the region involved in GTP binding and the one involved in metal and protein interactions being more rigid and more flexible, respectively. EPR also revealed that concomitant addition of both Ni(II) and GTP is necessary for a structural transition in these two parts of the protein, located on opposite sides of the tertiary structure, with a shift of the conformational equilibrium towards a more structured state. Differently, addition of either the metal ions or the nucleotide produces only minor perturbations of the conformational equilibrium, indicating that both ligands are necessary to exert a significant conformational response. These observations suggest that binding of GTP in its pocket is propagated, along the protein backbone, to the metal binding site where Ni(II) is bound, and vice versa. The induced shift of the conformational ensemble of UreG likely regulates the protein function, possibly allowing the protein to shuttle Ni(II) ions from UreE to the UreD_2_-UreF_2_ complex and, eventually, to urease.

Overall, the present work represents an important contribution for the characterization of the dynamics of UreG and its role in the network of the urease chaperone proteins. In the perspective to extend this original approach, involving SDSL-EPR spectroscopy, to the study of urease network directly inside bacterial cells, the results presented here provide important insights useful in the research on molecules with anti-bacterial activities to overcome anti-microbial resistance (AMR).

## Figures and Tables

**Table 1:** Thermodynamic parameters of Ni(II) titrations over *Hp*UreG and its variants, in the absence and in the presence of GTPγS.

Sample in the ITC Cell	N	K_a_ (M^−1^)	K_d_ (µM)	ΔH (kcal mol^−1^)	ΔS (cal mol^−1^K^-1^)
*Hp*UreG	2	1.38 ± 0.02 × 10^4^	72 ± 1	−5.90 ± 0.06	−0.86
C66A-*Hp*UreG	1	4.23 ± 0.02 × 10^3^	236 ± 1	−5.52 ± 0.01	−1.93
WT^prox^-*Hp*UreG	1	4.33 ± 0.04 × 104	23.0 ± 0.3	−4.99 ± 0.02	4.47
*Hp*UreG-GTPγS	HpUreG-Ni	3.45 ± 0.02 × 10^4^	30.0 ± 0.2	−7.14 ± 0.06	−4.99
	HpUreG_2_-Ni	1.40 ± 0.04 × 10^4^	71 ± 2	+11.2 ± 0.3	+56.77
C66A*Hp*UreG-GTPγS	C66AHpUreG-Ni	6.1 ± 0.8 × 10^3^	160 ± 20	−61 ± 7	−187
	C66AHpUreG_2_-Ni	4.5 ± 0.7 × 10^4^	22 ± 3	+99 ± 9	+353
WT^prox^-*Hp*UreG-GTPγS	WT^Prox^-HpUreG-Ni	4.20 ± 0.04 × 10^4^	23.8 ± 0.2	−2.63 ± 0.03	+12.3
	WT^Prox^-HpUreG_2_-Ni	3.8 ± 0.3 × 10^3^	260 ± 20	+9.1 ± 0.5	+46.8

**Table 2:** Electron Paramagnetic Resonance (EPR) spectra simulation parameters: rotational correlation times (τ_c_ in ns) and proportion (%) of the simulated multi-components of the spectra of *Hp*UreG variants under various conditions.

		Apo-Form		+Ni(II)		+Ni(II) and GTP	
Labeled Site	Component	Weight %	τc (ns)	Weight %	τc (ns)	Weight %	τc (ns)
C66^prox^	Fast	56	0.6	34	0.6	28	0.7
	Slow	44	2.4	66	2.4	72	3.4
C66^prox^	Fast	30	0.6	22	0.6	*19*	0.8
*+gly 30%*	Slow/Rigid ^*^	70	2.4	78	2.4	81 ^*^	4.4 ^*^
C48^prox^	Fast	37	0.6	39	0.6	21	0.6
	Rigid	63	4.9	61	4.9	79	4.9
C7^prox^	Fast	20	0.3	15	0.3	14	0.3
	Rigid	80	6.1	85	6.1	86	6.1

The symbol (*) indicates the “rigid” component.

## Supplementary Materials

The following can be found at https://www.mdpi.com/2218-273X/10/7/1062/s1: Supporting figures and tables as well as detailed descriptions of all experimental procedures, comprising EPR spectra simulations, site-directed mutagenesis, measurement of UreG GTPase activity, Size exclusion chromatography and light scattering data, are provided with the manuscript. Section S1: Supplementary figures (S1–S13), Section S2: CW spectra simulation with SimLabel program, Section S3: Methods summary.
