# Supplementary material for: Nickel and GTP Modulate Helicobacter pylori UreG Structural Flexibility
Source: Biomolecules. 2020 Jul 16;10(7):1062. doi: 10.3390/biom10071062 (PMC7408563; doi:10.3390/biom10071062)
Supplement: Supplementary file 1 [file biomolecules-10-01062-s001.pdf]

SUPPLEMENTARY DATA

Nickel and GTP modulate *Helicobacter pylori* UreG structural flexibility.

Annalisa Pierro,[1] Emilien Etienne,[1] Guillaume Gerbaud,[1] Bruno Guigliarelli,[1] Stefano Ciurli,[2] Valérie Belle,[1] Barbara Zambelli,[2,*] Elisabetta Mileo.[1,*]

[1] *Aix Marseille Univ, CNRS, Bioénergétique et Ingénierie des Protéines, IMM, Marseille, France.*
[2] *University of Bologna, Laboratory of Bioinorganic Chemistry, Department of Pharmacy and Biotechnology, Bologna, Italy.*

SECTION 1: Supplementary figures

SECTION 2: CW spectra simulation with SimLabel program

SECTION 3: Methods summary

SECTION 1: SUPPLEMENTARY FIGURES

Figure S1: MALDI-ToF analysis: **A**) *Hp*UreG**WT** labeled with proxyl in 1, 2 and 3 sites respectively; **B**) *Hp*UreG **C48** (Cys7Ala/Cys66Ala) unlabeled (red) and labeled with proxyl (blue), **C**) *Hp*UreG-**C66** (Cys7Ala/Cys48Ser) labeled with proxyl (green); **D**) *Hp*UreG **C7** (Cys48Ser/Cys66Ala) labeled with proxyl (black). Stars indicate artifacts due to matrix-adducts. The measured mass increments between the unlabeled and labeled protein are in good agreement with the expected value (235 Da). The error on the measurement is of +/- 5 Da.

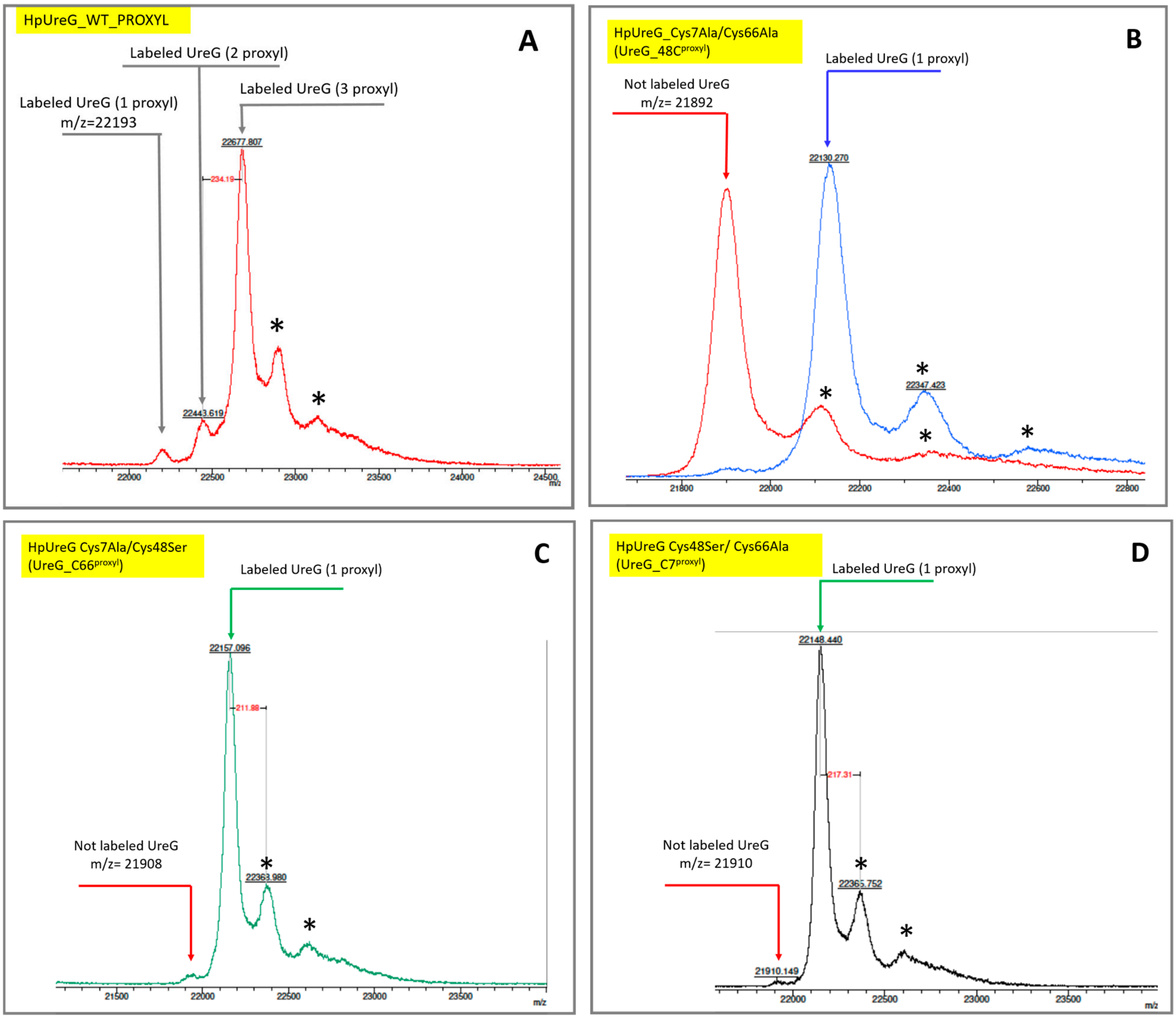

***MALDI-ToF MS Spectrometry** - Determination of Global Mass of unlabeled and labeled* UreG.
After the labeling procedure, mass analysis (MALDI-ToF) was performed to confirm the labeling. Expected mass increment resulting from one molecule of nitroxide grafted on the protein is of 235 Da for M-PROXYL nitroxide. Samples of ~80 pmol of unlabeled HpUreG and labeled HpUreG were prepared by dilution in 10 µL of 0,1 % of trifluoro-acetic acid (TFA) in water (v/v) before being spotted onto a MALDI target plate (1 µL). A saturated solution of sinapic acid matrix (1 µL) in 70 % acetonitrile/water, 0,1% TFA (v/v) was added. The global mass was measured on a MALDI-ToF mass spectrometer Microflex II from Bruker Daltonics in the range of 2000 to 65000 Da in a positive linear mode. External mass calibration was performed using the signals from the Protein standard I (Bruker Daltonics). The error on the measurement is of +/- 5 Da.

Figure S2: CD spectra of *Hp*UreG and its variants. CD spectra were recorded on a Jasco 815 CD spectrometer flushed with N2 using 0.1 cm thick quartz cells in Phosphate Buffer 10 mM pH 7,5 NaCl 150 at 25 °C. CD spectra were measured from 250 to 190 nm, at 20 nm/min and were averaged from 10 scans. Mean ellipticity values per residue ($[\theta]_{mrw,\lambda}$) were calculated as $[\theta]_{mrw,\lambda}$ = MRW × θ_λ /(10 × d × c), where MRW is the mean residue weight, θ_λ is the observed ellipticity (in deg) at wavelength λ, d is the pathlength (0.1 cm), and c is the protein concentration expressed in g/mL. Protein concentrations of 20 µM were used.

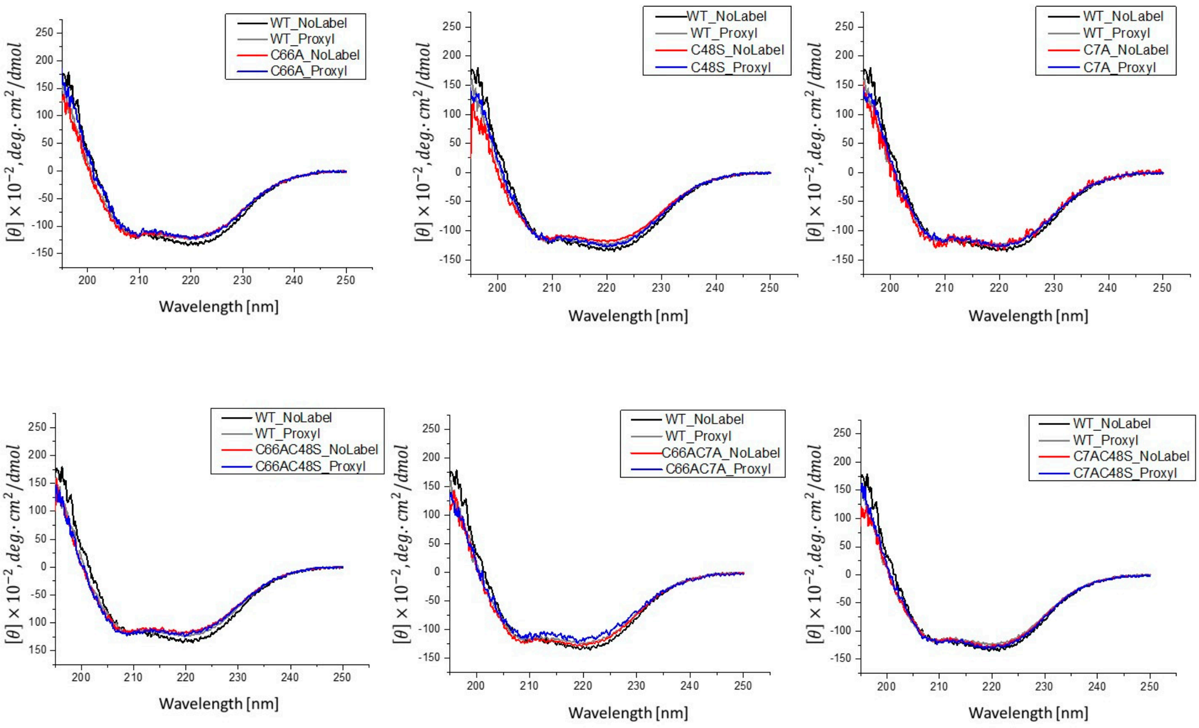

In all the panels showed above, the CD spectrum of the unlabeled protein (black line) is compared with that one labeled with Proxyl spin label (grey line), with one of the variant unlabeled (red line) and labeled blue line).

The CD spectra of *Hp*UreG mutants exhibit similar features as that one of the WT protein: the two characteristic minima at 208 and 220 nm confirm that the WT protein and the mutants keep the same content in α-helices. Thus, the mutation of one or two residues does not affect the overall secondary structure of the protein.

Figure S3: Light scattering analysis

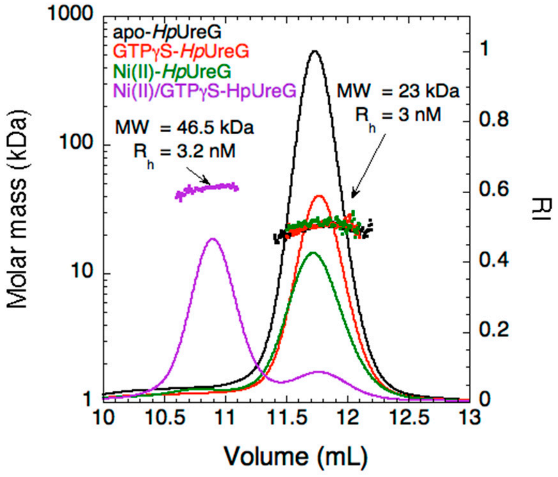

The light scattering data were collected using a Superdex-75 10/300 GL size-exclusion chromatography (SEC) column (GE Healthcare), connected to a differential refractometer (OptiLab Dsp, Wyatt technologies) and a static multi-angle laser light scattering (MALS) detector (Dawn Dsp, Wyatt technologies), as previously described.[4] HpUreG (160 μM in 100 μL), in the absence and in the presence of 400 μM $NiSO_4$ and/or 320 μM GTPγS, was injected into the SEC/MALS/RI system pre-equilibrated in 20 mM Tris, pH 8.0, 150 mM NaCl, 1 mM TCEP at a flow rate of 0.5 mL min^{-1}. Values of 1.33 for the refractive index of the solvent and of 0.185 mL g^{-1} for dn/dc were used. Data analysis was performed with the ASTRA software and a Rayleigh–Debye–Gans (RGD) light scattering model, according to manufacturer's instructions.

Figure S4: Superposition of experimental X-band CW EPR spectra of **C66^{prox} in Tris buffer** (black line) and **C66^{prox} +glycerol 30%** (red line).

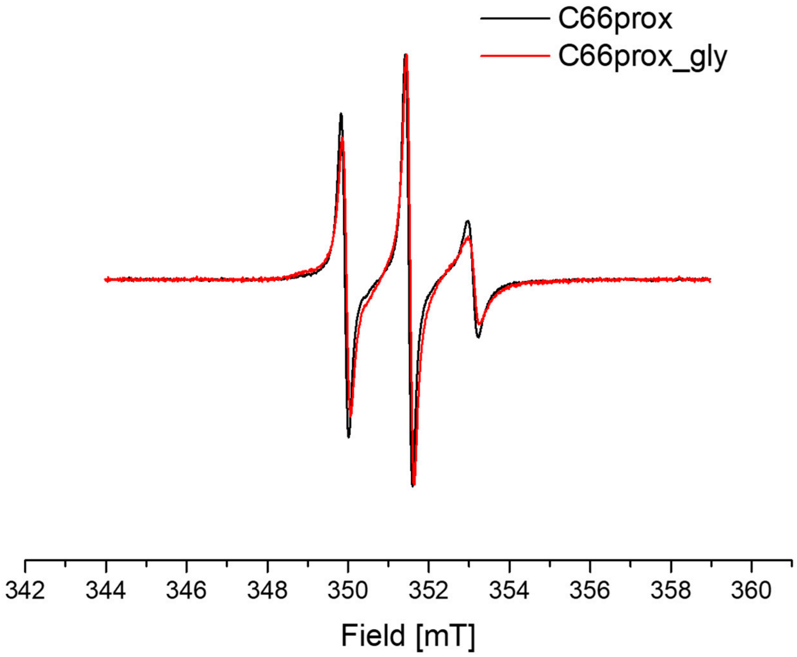

Figure S5: A) Experimental EPR spectrum of *Sp*UreG^{prox} (black line) and its simulation (red line); B) Parameters resulting from the simulation of *Sp*UreG^{prox} spectrum.

A)

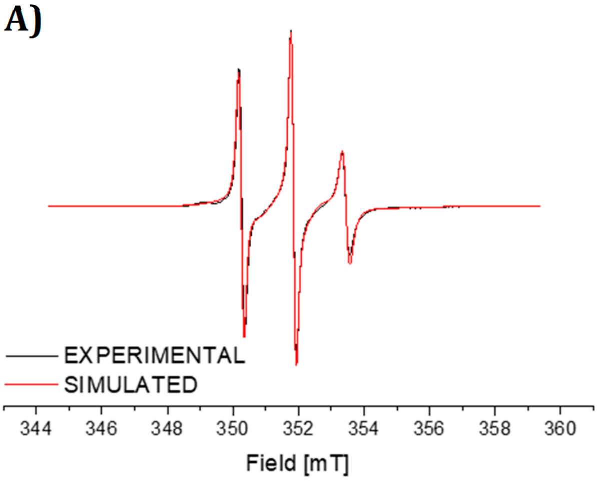

B)

SpUreG ^{prox}	Component 1	Component 2
τ_c	0,34ns	3,6ns
%	47%	53%
g (x, y, z)	2,00807 2,00610 2,00220	2,00663 2,00610 2,00220
a_N (mT)	0,36 0,36 4, 90	0,53 0,53 3,71
a_N iso (mT)	1,60	1,59
Lw (mT)	G:0,12 L:0,04	G:0,14 L:0,03

Figure S6: *In-silico* labeling of *Hp*UreG WT using MMM software.[1] A) HpUreG monomer from pdb 4HI0, proxyl rotamers are showed in gold, GDP is showed in pink. B) Number of rotamers attached according to MMM analysis.

A

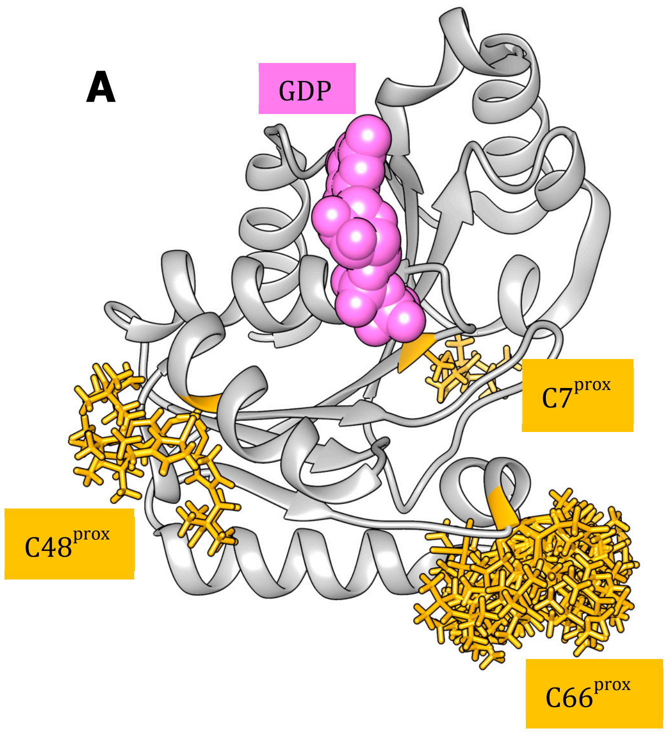

B

Residue	Number of rotamers
**C66**	25 ± 1
**C7**	1
**C48**	4

Figure S7: Experimental X-Band CW EPR spectra of double labeled HpUreG variants in 20 mM TrisHCl buffer pH 8, 150 mM NaCl at 25 °C.

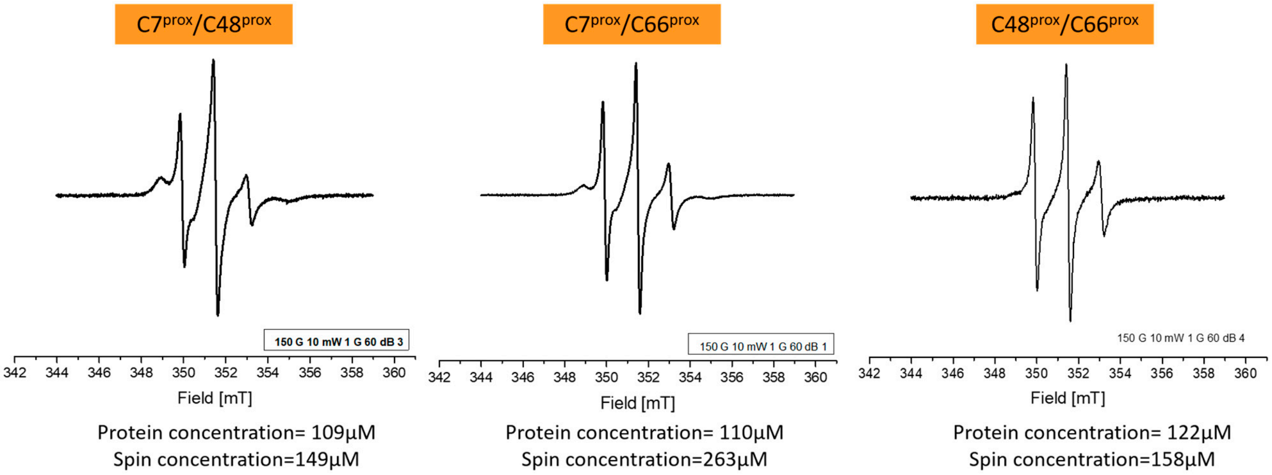

Figure S8: Validation of distance distributions obtained by DEER.
Distance distributions obtained from Tikhonov regularizations were validated using DEERNet, a validation tool of DeerAnalysis2019. The error bars (gray lines) are the full variation of the probability of given distances.

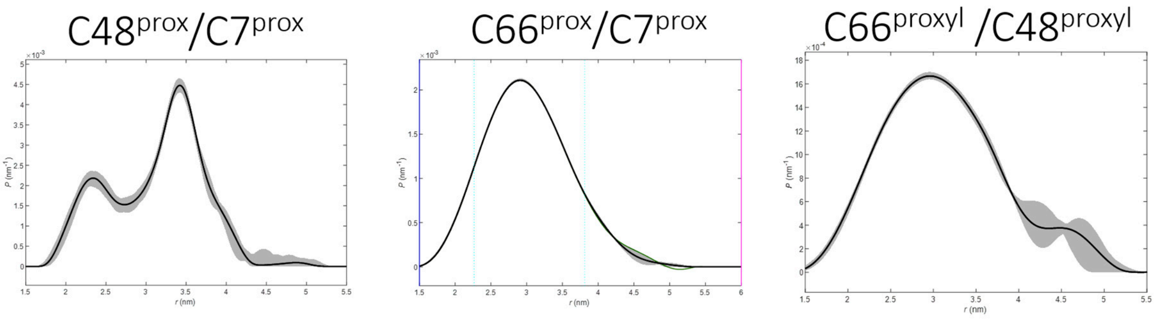

Figure S9: GTP effect on X-band CW EPR spectra of single labeled *Hp*UreG at room temperature: A) C7^{proxyl}; B) C48^{proxyl}; C) C66^{proxyl}. The apo-form spectra (black line) are compared with spectra recorded in presence of GTP 3mM (red line). Spectra were normalized by the maximum value.
For all the variants the concentration is 50μM in 20 mM TrisHCl buffer pH 8, 150 mM NaCl at 25 °C.

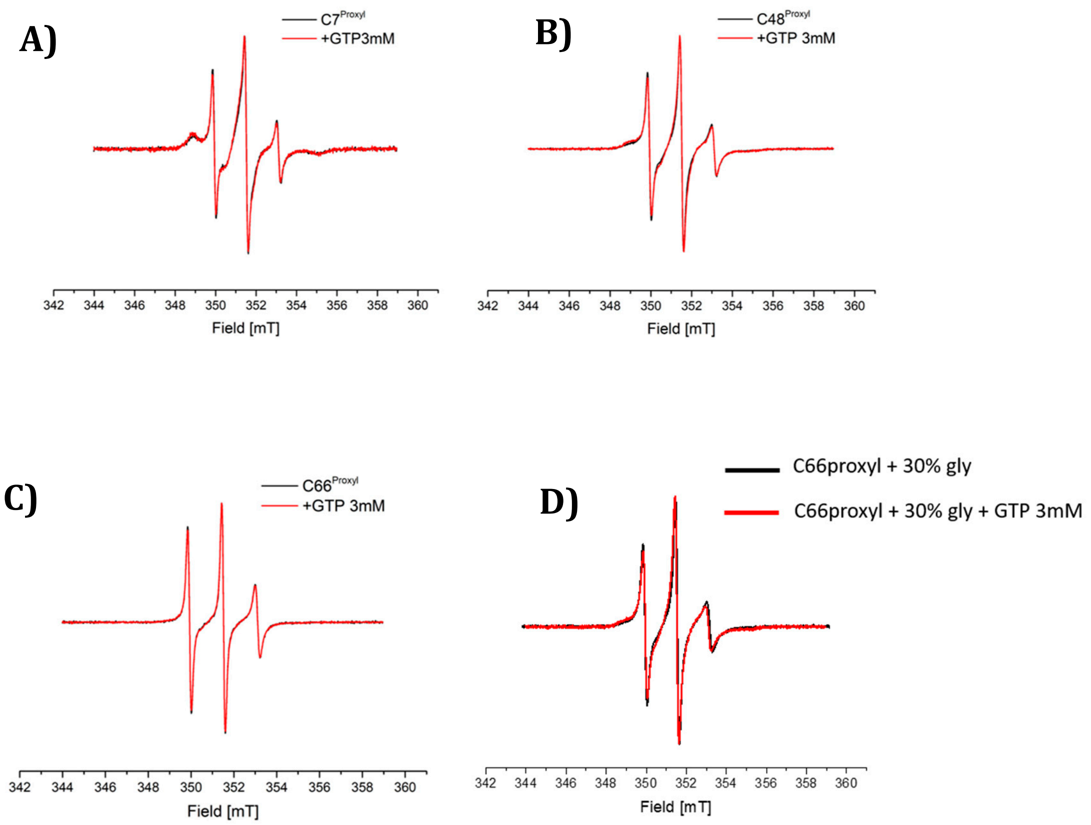

Figure S10: GDP effect on X-band CW EPR spectra of HpUreG-C48^{proxyl} at room temperature: A) The apo-form spectrum (black line) is compared with that one recorded in presence of GDP 3 mM (red line). B) The apo-form spectrum (black line) is compared with that one recorded in presence of GTP 3 mM and Ni(II) 2.5 mM (red line). Protein concentration was 50µM in 20 mM TrisHCl buffer pH 8, 150 mM NaCl at 25 °C. Spectra were normalized by the maximum value.

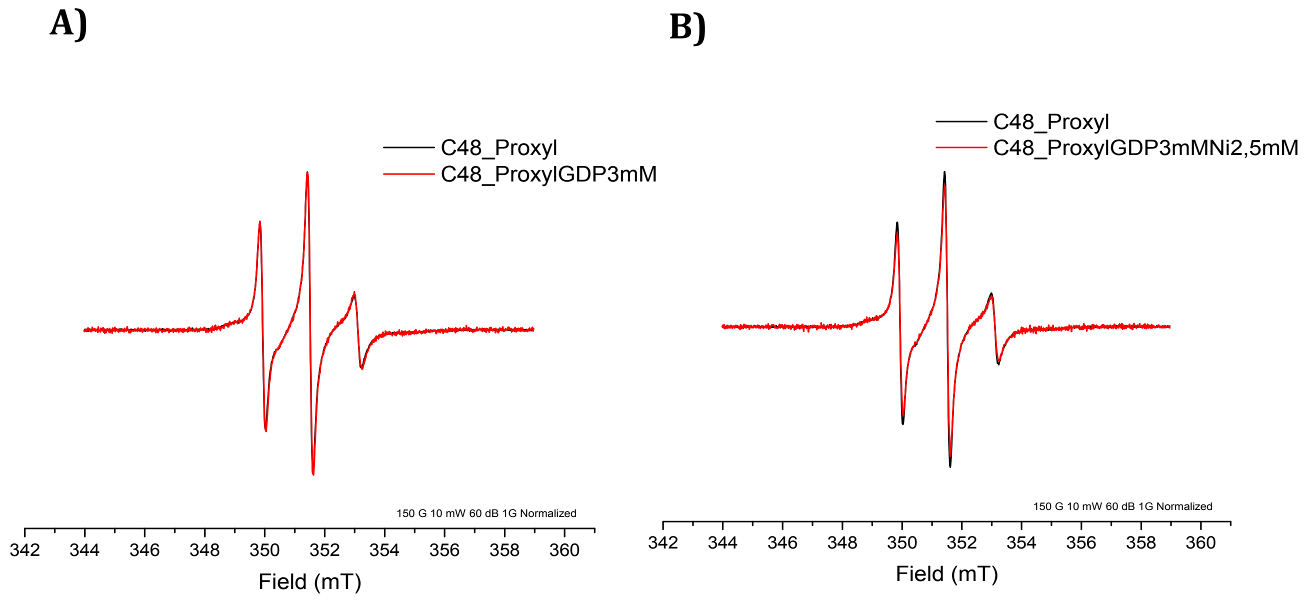

Figure S11. Mg^{2+} effect on *Hp*UreG wild type labeled with MA-Proxyl on the three Cys residues.
Black trace: X-band CW EPR spectrum of 50µM of protein in 20mM Tris buffer, NaCl 150mM, GTP 1mM.
Red trace: X-band CW EPR spectrum of 50µM of protein in 20mM Tris buffer, NaCl 150mM, GTP 1mM + $MgSO_4$ 1mM.
Spectra were normalized by the maximum value.

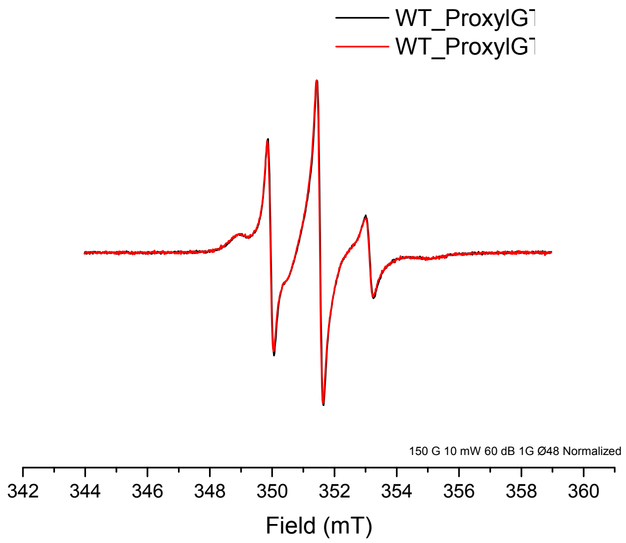

Figure S12: Effect of concomitant addition of 3mM GTP and 2.5mM Ni(II) on HpUreG-C48^{prox}/C66^{prox} + 30% glycerol. Room temperature CW EPPR spectra: the apo-form spectrum (black line) is compared with the one recorded in presence of GDP 3 mM and Ni(II) 2.5 mM (red line Protein concentration was 50μM in 20 mM TrisHCl buffer pH 8, 150 mM NaCl at 25 °C.
Spectra were normalized by the maximum value.

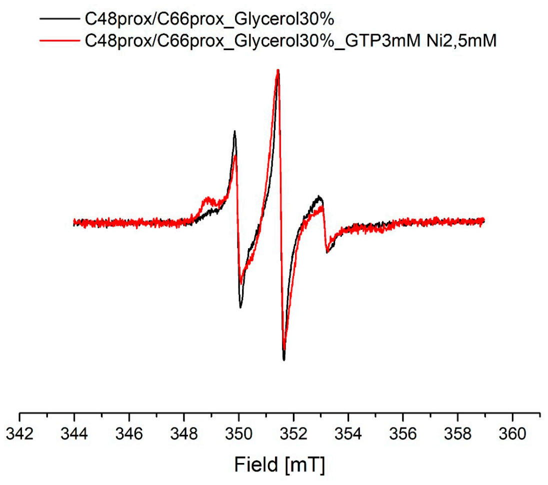

Figure S13: Decay curves showing the relaxation properties of C66^{proxyl} in absence *(black line)* and in presence of Ni^{2+} ions *(red line)* in Tris 20 mM, Nacl 150 mM.

A) Electron spin echo decay curve
B) Echo field sweep

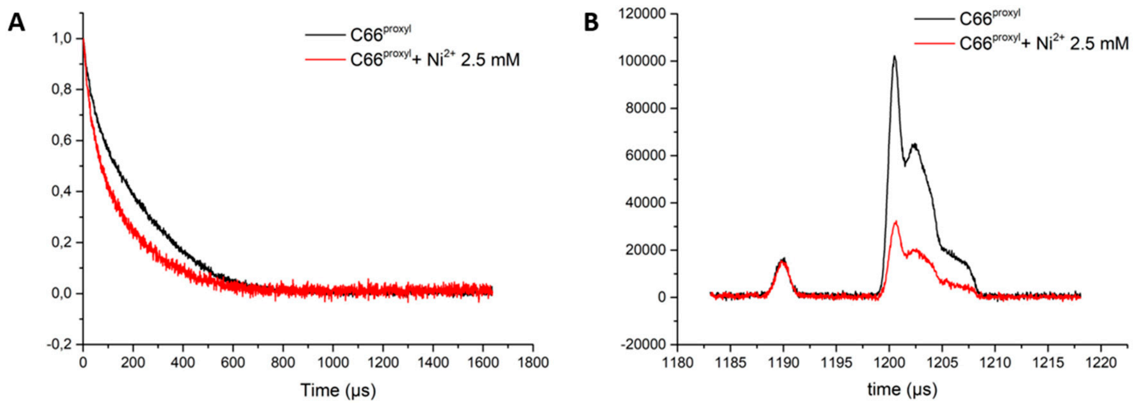

SECTION 2: CW spectra simulation with SimLabel program.

Experimental EPR spectra of *Hp*UreG variants labeled with spin label MA-Proxyl. Simulations were performed with SimLabel program (a GUI of EasySpin). All panels "A" show the simulated spectra (magenta line) superimposed on the experimental ones (black line). All panels "B" show the components required to obtain the best fits. The tables on the right side indicate all the parameters used for simulations.
In the tables: "tcorr"= correlation time; "%"= component weight.

*Hp*UreG C7^{prox} apo

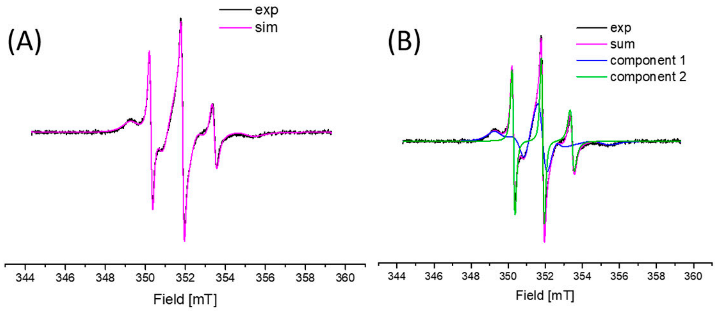

	sharp	broad
tcorr	0,34ns	6.2ns
%	20%	80%
g	2,00870 2,00610 2,00220	2,00834 2,00610 2,00220
aN (mT)	0,36 0,36 4,05	0,54 0,54 3,70
aN_{iso} (mT)	1,59	1,59
Lw (mT)	G: 0,10 L:0,08	G:0,02 L:0,01

*Hp*UreG C7^{prox} apo + Ni(II) 2.5 mM

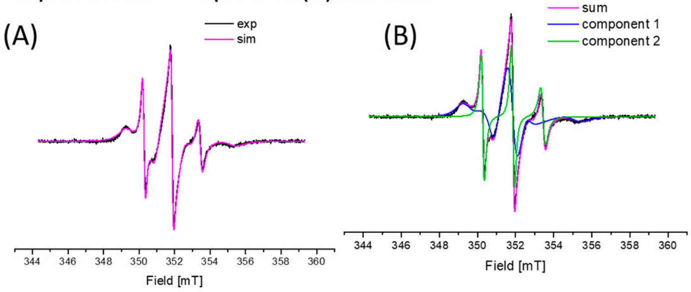

	sharp	broad
tcorr	0,34ns	6.2ns
%	15%	85%
g	2,00870 2,00610 2,00220	2,00834 2,00610 2,00220
aN (mT)	0,36 0,36 4,04	0,55 0,55 3,703
aN_{iso} (mT)	1,59	1,61
Lw (mT)	G: 0,13 L:0,05	G:0,12 L:0,02

*Hp*UreG C7^{prox} apo + Ni(II) 2.5 mM + GTP 3mM

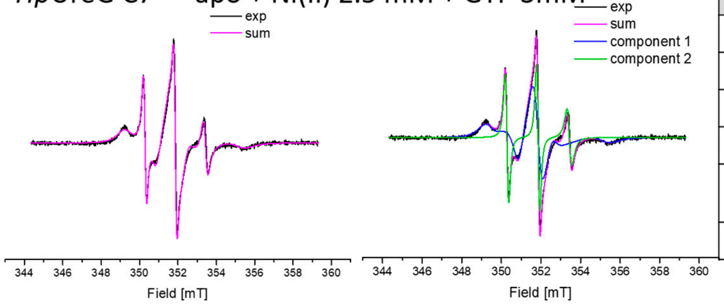

	sharp	broad
tcorr	0,34ns	6.2ns
%	16%	84%
g	2,00870 2,00610 2,00220	2,00834 2,00610 2,00220
aN (mT)	0,36 0,36 4,04	0,54 0,54 3,73
aN_{iso} (mT)	1,59	1,60
Lw (mT)	G: 0,11 L:0,09	G:0,06 L:0,01

*Hp*UreG C48^{prox} apo

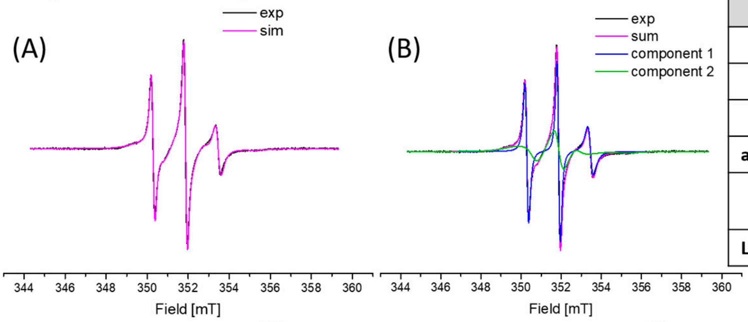

	sharp	broad
**tcorr**	0,62ns	4,1ns
**%**	37%	63%
**g**	2,00902 2,00510 2,00267	2,00760 2,00610 2,00220
**aN (mT)**	0,47 0,47 3,84	0,52 0,52 3,74
**aN_{iso} (mT)**	1,60	1,60
**Lw (mT)**	G: 0,11 L:0.02	G:0,04 L:0,01

*Hp*UreG C48^{prox} apo + Ni(II) 2.5 mM

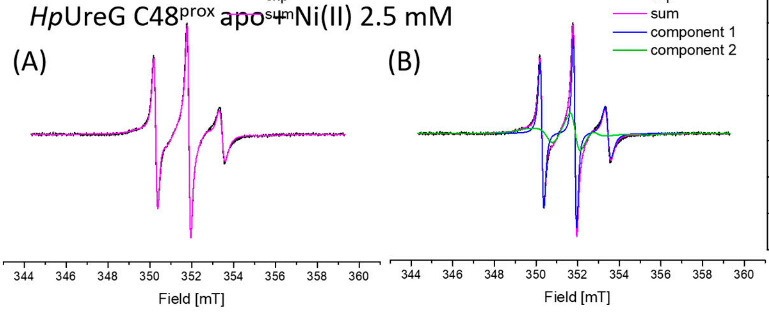

	sharp	broad
**tcorr**	0,62ns	4,1ns
**%**	39%	61%
**g**	2,00902 2,00510 2,00267	2,00760 2,00610 2,00220
**aN (mT)**	0,47 0,47 3,84	0,52 0,52 3,74
**aN_{iso} (mT)**	1,60	1,60
**Lw (mT)**	G: 0,11 L:0.02	G:0,04 L:0,01

*Hp*UreG C48^{prox} apo + Ni(II) 2.5 Mm + GTP 3mM

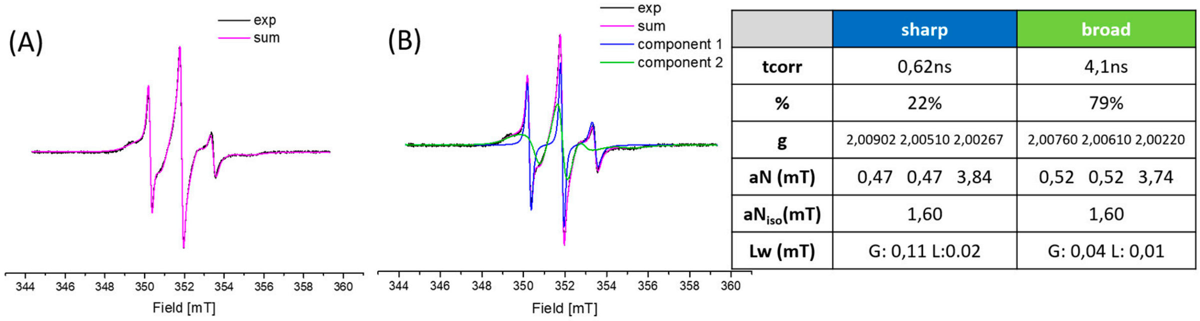

	sharp	broad
**tcorr**	0,62ns	4,1ns
**%**	22%	79%
**g**	2,00902 2,00510 2,00267	2,00760 2,00610 2,00220
**aN (mT)**	0,47 0,47 3,84	0,52 0,52 3,74
**aN_{iso}(mT)**	1,60	1,60
**Lw (mT)**	G: 0,11 L:0.02	G: 0,04 L: 0,01

*Hp*UreG C66^{prox} apo

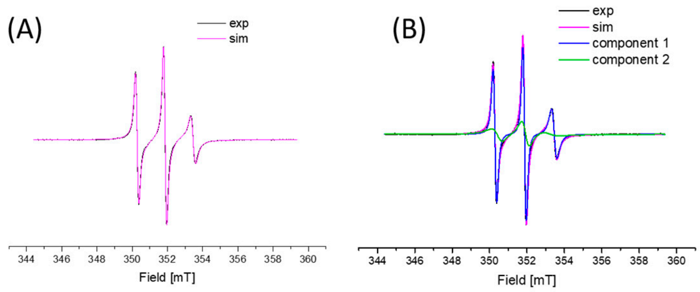

	sharp	broad
tcorr	0.56ns	2.4ns
%	56%	44%
g	2,00767 2,00610 2,00220	2,00735 2,00610 2,00220
aN (mT)	0.57 0.57 3.66	0,49 0,49 3,78
aN_{iso} (mT)	1,60	1.59
Lw (mT)	G: 0.13 L: 0.01	G:0.05 L: 0.01

*Hp*UreG C66^{prox} apo + Ni(II) 2.5 mM

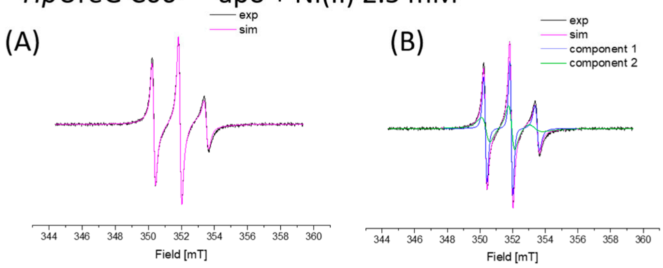

	sharp	broad
tcorr	0.56ns	2.4ns
%	*34%*	*66%*
g	2.00838 2.00510 2.00267	2.00701 2.00610 2.00220
aN (mT)	0.50 0.50 3.78	0.56 0.56 3.78
aN_{iso} (mT)	1.60	1.63
Lw (mT)	G:0.16 L:0.01	G: 0.12 L: 0.04

*Hp*UreG C66^{prox} apo + Ni(II) 2.5 mM + GTP 3mM

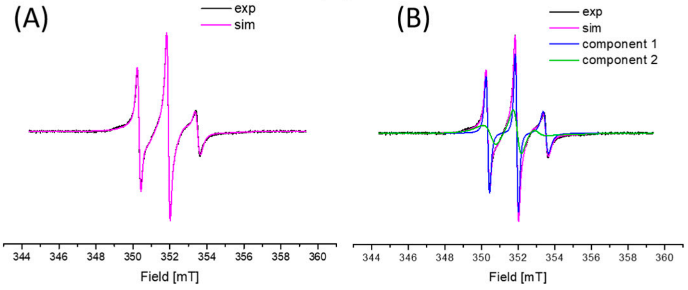

	sharp	broad
tcorr	0.68 ns	3.41 ns
%	*34%*	*66%*
g	2.00831 2.00510 2.00267	2.00679 2.00610 2.00220
aN (mT)	0.50 0.50 3.78	0.54 0.54 3.68
aN_{iso} (mT)	1.60	1.58
Lw (mT)	G:0.13 L:0.01	G: 0.16 L: 0.01

*Hp*UreG C66^{prox} apo in 30% glycerol

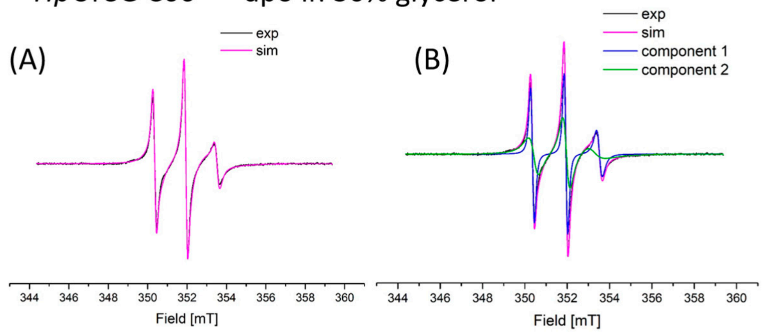

	sharp	broad
tcorr	0,56ns	2,4ns
%	30%	70%
g	2,00875 2,00510 2,00220	2,00702 2,00610 2,00220
aN (mT)	0,49 0,49 3,80	0,52 0,52 3,75
aN_{iso} (mT)	1,59	1,60
Lw (mT)	G: 0,13 L:0.01	G:0,03

*Hp*UreG C48^{prox} apo + Ni(II) 2.5 mM in 30% glycerol

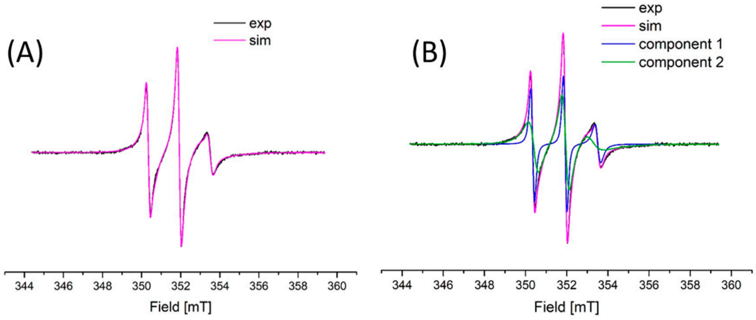

	sharp	broad
tcorr	0,56ns	2,4ns
%	22%	78%
g	2,00910 2,00510 2,00220	2,00732 2,00610 2,00220
aN (mT)	0,45 0,45 3,88	0,52 0,52 3,80
aN_{iso} (mT)	1,59	1,61
Lw (mT)	G: 0,13 L:0.01	G:0,03

*Hp*UreG C66^{prox} Ni(II) 2,5mM + GTP 3mM in 30% glycerol

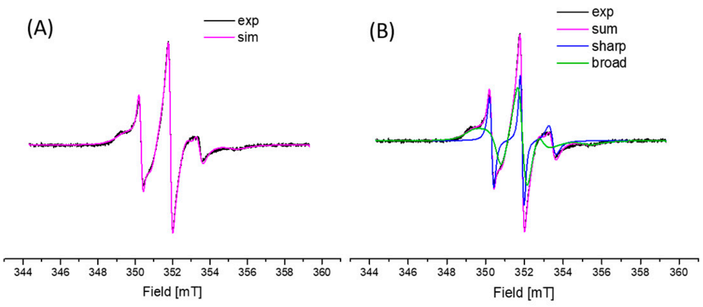

*Hp*UreG C66^{prox} +Gly30% GTP 2mM Ni 2.5mM	sharp	broad
**tcorr**	0,76 ns	4,41 ns
**%**	19	81
**g**	2,00941 2,00510 2,00267	2,00795 2,00523 2,00220
**aN (mT)**	0.36 0.36 4.04	0.55 0.55 3.81
**aN iso (mT)**	1.59	1.64
**Lw (mT)**	G :0.11 L :0	G :0.03 L :0.0

SECTION 3: Methods summary

Site-directed mutagenesis

The pairs of mutagenic primers were designed as following:

(C7A)
ATATACCATGGTAAAAATTGGAGTT**GC**TGGTCCTGTAGGAAGCG,
CGCTTCCTACAGGACCAGCAACTCC**CG**TTTTTACCATGGTATAT

(C48S)
AGAAGACGCAGAGTTTATG**T**GTAAAAATTCGGTGATGCC
GGCATCACCGAATTTTTAC**T**CATAAACTCTGCGTCTTCT

(C66A).
CGTAGAAACAGGAGG**C**GCTCCGCACACGGCTATT,
AATAGCCGTGTGCGG**A**GCGCCTCCTGTTTCTACG

At the end, all the mutations were verified by nucleotide sequencing.

Measurement of GTPase activity.

GTP hydrolyzing activity was measured under different buffer conditions, as reported in the Results section, using the SensoLyte® MG Phosphate Assay Kit (AnaSpec), based on the colorimetric reaction involving malachite green reagent, molybdate and orthophosphate, as previously reported for other UreG proteins[5]. Each sample was prepared mixing reagents in order to obtain 20 μM of protein, 400 μM of GTP and 2 mM of MgSO4 in a final volume of 250 μL of buffer. The reaction mixture (RM) was incubated for 2 hours at 37 °C. Every 30 minutes, 40 μL from the RM were incubated with 40 μL of Malachite Green Mix for 10 minutes in a final volume of 300 μL of buffer.

After incubation, the absorbance at 600 nm was recorded. The activity of the above-mentioned HpUreG was measured also in presence of 2 mM of NiSO4. All the experiments were reproduced two times before estimate the K_{cat} values Phosphate concentration was determined measuring the absorbance of the solution after 20 minutes of incubation, according to a calibration curve performed using phosphate standard solutions.

SIZE EXCLUSION CHROMATOGRAPHY AND LIGHT SCATTERING

The molecular mass and hydrodynamic radius of HpUreG in solution, in the absence and in the presence of Ni(II) and/or GTPϒS, were determined using a combination of size exclusion chromatography (SEC), multiple angle light scattering (MALS), and quasi-elastic light scattering (QELS). The proteins (100 μL, 160 μM) was loaded onto a Superdex-75 10 300 GL column (GE Health- care), and eluted in 20 mM Tris buffer pH 8.0, containing 150 mM NaCl and 1 mM TCEP and (when indicated) 400 μM NiSO4 and/or 320 μM GTPϒS, at a flow rate of 0.6 mL min−1. The column was connected downstream to a multiangle laser light (690.0 nm) scattering (MALS) DAWN EOS photometer and to a 90° angle Quasi-elastic (dynamic) light scattering (QELS) device (Wyatt Technology). The concentration of the eluted protein was determined using a refractive index detector (Optilab DSP, Wyatt). Values of 0.185 mL g−1 for the refractive index increment (dn/dc) and of 1.330 for the solvent refractive index were used. Molecular weights were determined from a Zimm plot, using the Zimm equation (Zimm, 1948) with a fitting degree of one. Data were analyzed using the Astra 4.90.07 software (Wyatt Technology), following the manufacturer's indications.

References.

1. Jeschke, G., MMM: A toolbox for integrative structure modeling. *Protein science : a publication of the Protein Society* **2018,** *27* (1), 76-85.

2. Etienne, E.; Le Breton, N.; Martinho, M.; Mileo, E.; Belle, V., SimLabel: a graphical user interface to simulate continuous wave EPR spectra from site-directed spin labeling experiments. *Magnetic resonance in chemistry : MRC* **2017,** *55* (8), 714-719.

3. Stoll, S.; Schweiger, A., EasySpin, a comprehensive software package for spectral simulation and analysis in EPR. *Journal of magnetic resonance (San Diego, Calif. : 1997)* **2006,** *178* (1), 42-55.

4. D'Urzo, A.; Santambrogio, C.; Grandori, R.; Ciurli, S.; Zambelli, B., The conformational response to Zn(II) and Ni(II) binding of Sporosarcina pasteurii UreG, an intrinsically disordered GTPase. *J Biol Inorg Chem* **2014,** *19* (8), 1341-54.

5. Miraula, M.; Ciurli, S.; Zambelli, B., Intrinsic disorder and metal binding in UreG proteins from Archae hyperthermophiles: GTPase enzymes involved in the activation of Ni(II) dependent urease. *J Biol Inorg Chem* **2015,** *20* (4), 739-55.
